# 3D Printed Nitrogen‐Doped Thick Carbon Architectures for Supercapacitor: Ink Rheology and Electrochemical Performance

**DOI:** 10.1002/advs.202206320

**Published:** 2023-02-07

**Authors:** Guoqiang Zhou, Mei‐Chun Li, Chaozheng Liu, Chuhang Liu, Zhenglin Li, Changtong Mei

**Affiliations:** ^1^ Co‐Innovation Center of Efficient Processing and Utilization of Forest Resources College of Materials Science and Engineering Nanjing Forestry University Nanjing 210000 China; ^2^ School of Petroleum Engineering China University of Petroleum (East China) Qingdao Shandong 266580 China

**Keywords:** 3D printing, areal capacitance, rheology, supercapacitors, thick carbon electrodes

## Abstract

The 3D printing technique offers huge opportunities for customized thick‐electrode designs with high loading densities to enhance the area capacity in a limited space. However, key challenges remain in formulating 3D printable inks with exceptional rheological performance and facilitating electronic/ion transport in thick bulk electrodes. Herein, a hybrid ink consisting of woody‐derived cellulose nanofibers (CNFs), multiwalled carbon nanotubes (MWCNTs), and urea is formulated for the 3D printing nitrogen‐doped thick electrodes, in which CNFs serve as both dispersing and thickening agents for MWCNTs, whereas urea acts as a doping agent. By systematically tailoring the concentration‐dependent rheological performance and 3D printing process of the ink, a variety of gel architectures with high geometric accuracy and superior shape fidelity are successfully printed. The as‐printed gel architecture is then transformed into a nitrogen‐doped carbon block with a hierarchical porous structure and superior electrochemical performance after freeze‐drying and annealing treatments. Furthermore, a quasi‐solid‐state symmetric supercapacitor assembled with two interdigitated carbon blocks obtained by a 3D printing technique combined with a nitrogen‐doping strategy delivers an energy density of 0.10 mWh cm^−2^ at 0.56 mW cm^−2^. This work provides guidance for the formulation of the printable ink used for 3D printing of high‐performance thick carbon electrodes.

## Graphical abstract

A 3D printable CMU ink consisting of CNFs, MWCNTs, and urea is successfully developed by rational tailoring the concentration‐dependent rheological performance of inks and optimization of the 3D printing parameters. The N‐doped, hierarchically porous, thick carbon architectures can be achieved by 3D printing, freeze‐drying, and annealing approach, which hold the great potential in customized electrochemical energy storage device.

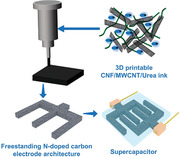

## 1 Introduction

With the continuous innovation and breakthrough of advanced intelligent and portable electronic products, there is an urgent need to develop high‐performance miniaturized electrochemical energy storage (EES) devices for their use.^[^ ^1^ ^]^ Supercapacitors (SC) have the merits of fast charge–discharge rate, large power capacity, and long cycle life and are considered one of the most promising EES devices that can be applied to power electronics.^[^ ^2^ ^]^ Conventional supercapacitors are routinely constructed in ultrathin forms with a low loading of active substances, resulting in unsatisfactory areal capacity, which limits the ability of the device to provide sufficient energy output within the restricted area to meet practical requirements.^[^ ^3^ ^]^ The active material coverage should be increased within the effective area or along the neglected height direction to address this issue. To this end, many scientists have attempted to prepare 3D free‐standing electrodes for SC.^[^ ^4^ ^]^ Nevertheless, developing 3D electrodes with customizable architectures remains a crucial challenge because traditional fabrication approaches, such as freeze‐casting, template assistance, and self‐assembly, are less precisely controlled techniques, and it is difficult to realize high geometric accuracy for specific uses.^[^ ^5^ ^]^

Direct ink writing (DIW), an extrusion subset of 3D printing technology, has been widely applied in manufacturing arbitrarily designated 3D structures used in various fields, including EES devices.^[^ ^6^ ^]^ This bottom‐up additive manufacturing technology can transform gel ink into a complicated architecture with high geometric accuracy in a layer‐by‐layer deposition manner based on a designed digital model.^[^ ^7^ ^]^ In other words, the DIW technique can control the height of the electrode architecture to increase the loading density of the active material within a limited footprint, thus improving the areal capacity. In addition, the desirable freedom in material selection for the formulation of inks enables DIW to grant exceptional edge in building different advanced functional 3D electrodes.^[^ ^8^ ^]^ However, the rheological behaviors of gel inks, which involve superior shear‐thinning ability, excellent viscoelasticity, and distinguished thixotropy, are essential for DIW because they directly determine the extrudability of inks, as well as the shape fidelity and structural stability of the architectures after printing.^[^ ^9^ ^]^ Also, rational printing parameters (e.g., pressure and speed) are necessary to achieve a high‐precision structure.^[^ ^10^ ^]^ Hence, formulating printable ink with outstanding rheological behavior and optimizing its corresponding printing process are prerequisites for structural customizability. In addition, commonly used carbon‐based electrode materials for SC, such as porous carbon, graphene oxide, and carbon nanotubes, usually exhibit limited dispersing capability and strong aggregating propensity,^[^ ^11^ ^]^ which undoubtedly cause inhomogeneity of ink that could lead to blockage of the nozzle during DIW and uneven loading of the active substance in the final electrode. Consequently, the uniform dispersion of active materials in the gel ink is a non‐negligible important factor in ensuring the superior 3D printability of the gel ink and the stable electrochemical performance of the printed electrode.

Although the 3D printing technique can easily increase the loading density of active materials in the 3D electrode, the diffusion of the electrolyte along complex routes in a tortuous manner within the 3D‐printed thick bulk electrode leads to sluggish electrochemical reaction kinetics, which limits the electrochemical performance at large charge–discharge rates.^[^ ^12^ ^]^ For 3D‐printed carbon‐based thick electrodes, constructing a hierarchical porous structure inside the electrode, heteroatomic doping in carbonaceous structures, and modifying the surface chemistry of the electrode material have been reported as effective strategies to improve the charge storage kinetics.^[^ ^8^, ^13^ ^]^ In particular, heteroatom doping can be used to adjust the fundamental characteristics of the active material to improve its electrochemical reactivity and charge storage kinetics.^[^ ^14^ ^]^ Typically, substitutional doping with nitrogen (N) elements into carbon surfaces has been demonstrated to enhance the wettability, conductivity, and structural defects of 3D‐printed carbon‐based architecture, thereby contributing to improved electrochemical performance.^[^ ^13a^ ^]^ These previous studies confirmed that the N‐doped strategy is promising for manufacturing high‐performance electrodes for EES devices. Herein, we combine the 3D printing technique with an N‐doping strategy to realize a customized design of thick electrodes and improve the charge storage kinetics, thus endowing the 3D‐printed thick electrode with desirable capacitive and rate performance. To achieve these goals, the formulation and rheological performance of inks containing N resources require comprehensive studies to ensure 3D printability. Additionally, it is important to take full advantage of the 3D printing technique to customize block electrodes in different shapes for SC devices.

**Figure 1:**
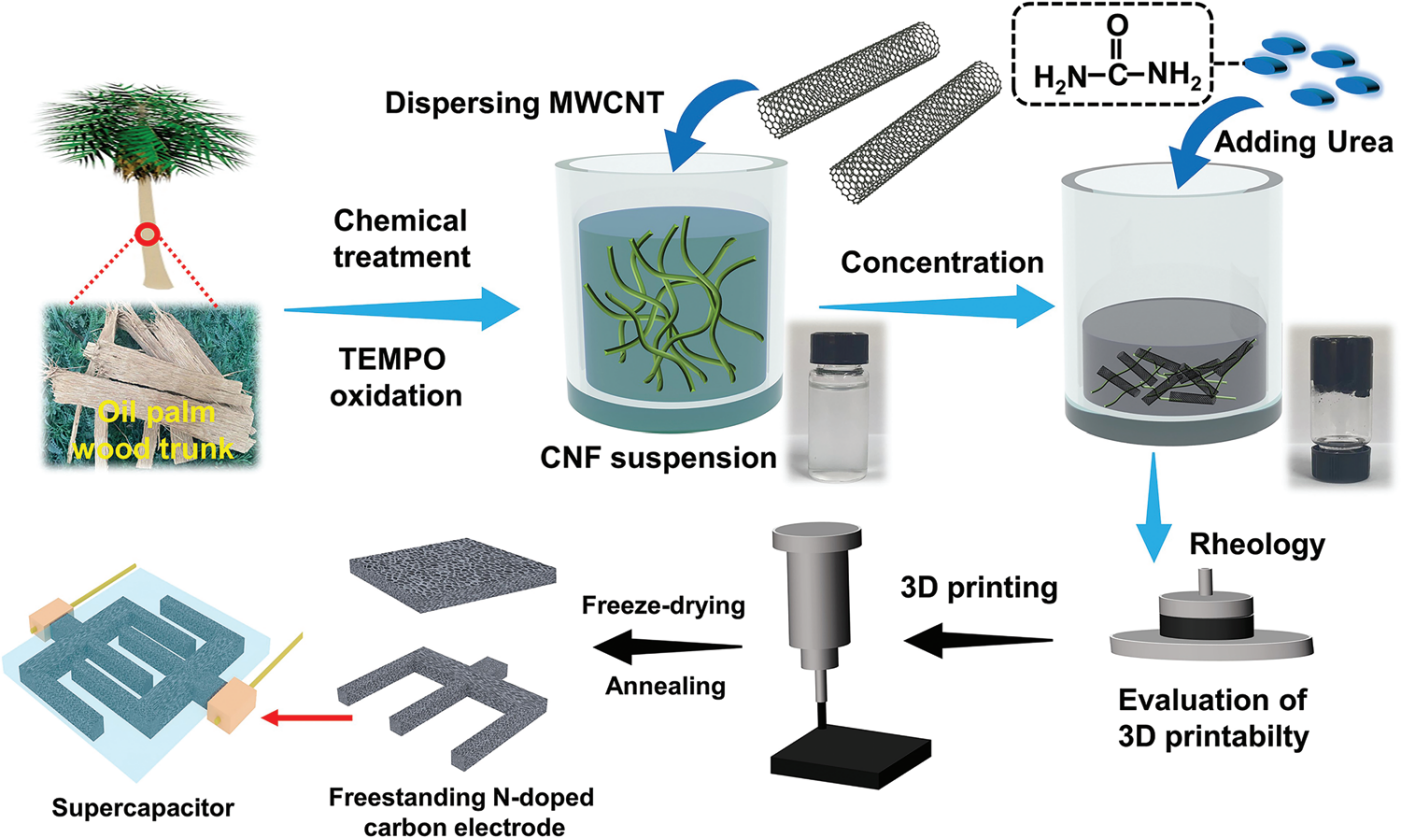
A schematic diagram illustrating the preparation of gel ink, the evaluation of 3D printability of gel ink by rheological measurements, the preparation of free‐standing, hierarchically porous N‐doped carbon architecture by 3D printing, freeze‐drying, and annealing treatments, and the fabrication of a quasi‐solid‐state symmetrical supercapacitor using two 3D‐printed N‐doped carbon electrodes.

In this study, woody‐derived cellulose nanofibers (CNFs) with carboxylate groups on the surface were utilized to disperse carboxylated multiwalled carbon nanotubes (MWCNTs) in an aqueous solution, followed by concentration, homogenization, and addition of urea to formulate a homogeneous, viscoelastic gel ink (**Figure** **1**). The concentration‐dependent rheological properties and extrudability of gel inks were systematically investigated to evaluate their 3D printability. Subsequent 3D printing of the optimized CNF/MWCNT/urea ink under rational printing parameters generated a variety of customizable 3D gel architectures with high geometric accuracy and structural stability. After freeze‐drying and annealing, a free‐standing N‐doped carbon block with a hierarchical porous structure was successfully obtained. Note that the in situ N‐doping effect induced by the decomposition of urea during the annealing process endowed the 3D‐printed carbon block with structural defects, high conductivity, and superb wettability. Hence, the 3D‐printed thick carbon block with 6 layers (i.e., ≈2.4 mm in height) showed excellent areal capacitance of 4.2 F cm^−2^ (17.3 F cm^−3^) at 2 mA cm^−2^ and superior rate performance (2.81 F cm^−2^ at 30 mA cm^−2^). Moreover, to prove the structural customizability of the 3D printing technique, a quasi‐solid‐state symmetric supercapacitor (QSSC) was constructed based on two 3D‐printed pectinate carbon architectures. The QSSC exhibited a favorable areal capacitance of 0.91 F cm^−2^ at 2 mA cm^−2^ (0.45 F cm^−2^ at 30 mA cm^−2^), good cycling stability with capacitance retention of 89% after 5000 charge–discharge cycles and reached an energy density of 0.10 mWh cm^−2^ at 0.56 mW cm^−2^.

## 2 Results and Discussion

An ideal ink for DIW 3D printing must coordinate various aspects, including the effective interaction between the ink components, good dispersion of each component in ink, and excellent rheological behavior. In particular, for the MWCNTs used in this study, their uniform dispersion is the primary guarantee for the formulation of stable and 3D printable ink. Indeed, dispersing MWCNT bundles into stable individual MWCNTs in highly concentrated 3D printing ink remains a challenge because the strong van der Waals forces between the MWCNTs would lead to severe agglomeration.^[^ ^15^ ^]^ This study used woody‐derived CNFs with negative surface charges to disperse MWCNTs in ink. The CNFs were isolated from oil palm wood by chemical treatment and 2,2,6,6‐Tetramethylpiperidine 1‐oxyl (TEMPO)‐mediated oxidation combined with ultrasonication, according to our previous work (Figure S1, Supporting Information).^[^ ^16^ ^]^ It is worth mentioning that the electrostatic repulsion forces induced by the surface electronegative carboxylic groups introduced during the TEMPO oxidation process endow the CNFs with excellent dispersibility in aqueous solutions.^[^ ^17^ ^]^ As a result, the CNF suspension at a concentration of 0.2 wt.% exhibits favorable colloid stability, as evidenced by its high zeta potential value of −39.17 mV (Figure S2, Supporting Information). A CNF/MWCNT hybrid suspension with a CNF/MWCNT mass ratio of 3/7 was prepared by adding MWCNTs to the CNF suspension, followed by high‐intensity ultrasound treatment. The CNF/MWCNT hybrid suspension (0.66 wt.%) has a high zeta potential value of −30.87 mV as well, and thus it exhibits high colloidal stability; that is, no sediment aggregates are observed even after standing for 24 h (Figures S2 and S3, Supporting Information). In contrast, severe aggregation of MWCNTs occurred in the absence of CNFs due to the strong van der Waals forces between the MWCNTs. The enhanced dispersion and colloidal stability of MWCNTs in CNF suspensions result from the electrostatic repulsive forces between the CNFs and steric hindrance between these two building blocks.^[^ ^18^ ^]^ In addition, the physical entanglement of the 1D CNFs and MWCNTs (Figure S4, Supporting Information) effectively prevented the aggregation of MWCNTs. With this dispersing strategy, >500 mL of a uniform CNF/MWCNT hybrid suspension can be easily achieved, suggesting its scalability and the merits of sustainability and environmental friendliness (Figure S5a, Supporting Information). The CNF/MWCNT gel was subsequently obtained by concentrating the CNF/MWCNT suspension to remove part of the water, and urea was added to the gel as the nitrogen source, followed by homogenization using a vacuum planetary high‐speed mixer. This significantly improved the uniform dispersion of the three components in the gel ink and eliminated most of the air bubbles from the gel ink. It is worth mentioning that highly hydrophilic absorbent beads were utilized to remove part of the water from the ink in the concentration process according to previous studies,^[^ ^8^, ^16^ ^]^ which is detailed in Experimental Section (Supporting Information). To investigate the effect of the concentration (i.e., solid content) on the rheological properties of inks and to evaluate the 3D printability, a series of CNF/MWCNT/urea (CMU) gel inks with various concentrations from 1 to 6 wt.% were successfully developed using the dispersing, concentrating, and homogenizing approaches.

**Figure 2:**
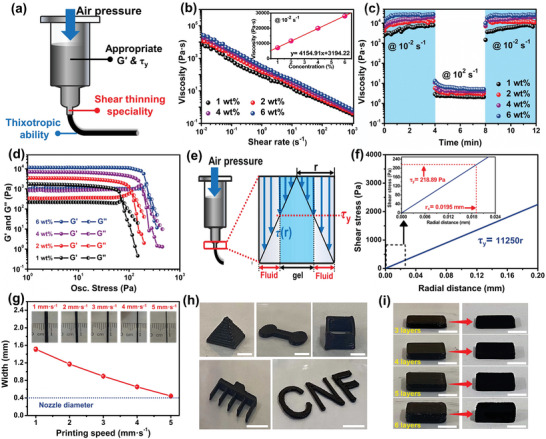
Rheological properties and 3D printability of the CMU gel inks: a) Schematic diagram of the rheological behaviors of ink required for DIW 3D printing. b) Viscosity as a function of shear rate, inset is the viscosity at the shear rate of 10^−2^ s^−1^. c) Viscosity evolution over time when alternatively applying the low (10^−2^ s^−1^) and high shear rates (10^2^ s^−1^). d) *G*′ and *G*″ as a function of oscillatory stress. e) Schematic diagram of shear stress driven by air pressure within the printed nozzle. f) Theoretical line of the shear stress inside the nozzle (*τ*_r_) as a function of radial distance (r), and the inset represents the determination of critical radius (*r*_c_) when *τ*_r_ is equal to the yield stress (*τ*_y_). g) The width of the extruded filaments under different printing speeds at the air pressure of 45 KPa; insets are the corresponding digital images of extruded filaments. h) Different 3D‐printed gel architectures using 6 wt.% ink, including pyramid (scale bar: 5 mm), dumbbell (scale bar: 5 mm), hollow cube (scale bar: 5 mm), pectinate structure (scale bar: 10 mm), and CNF font (scale bar: 10 mm). i) Digital images of printed blocks with different layers before and after freeze‐drying and annealing treatments. The scale bar is 5 mm.

For the DIW 3D printing technique, the rheological properties of the ink (e.g., shear‐thinning behavior, thixotropic capacity, and viscoelastic performance) play extremely important roles in 3D printability because they directly govern the extrudability of inks as well as the shape fidelity and geometrical accuracy of the 3D‐printed architectures.^[^ ^9a^ ^]^ In brief, a desirable 3D printable ink must hold: 1) a pronounced shear‐thinning specialty that allows the smooth extrusion of ink through the 3D printing nozzle under shear stress to avoid clogging; 2) excellent thixotropic capacity to ensure the rapid recovery of viscosity and viscoelasticity after printing; and 3) appropriate viscoelastic indexes, including storage modulus and yield stress, to ensure high geometrical accuracy of architectures and avoid structural collapse and deformation after printing (**Figure** **2**a). Therefore, a comprehensive characterization of the rheological properties of CMU gel inks, including the steady‐state shear viscosity (Figure 2b), thixotropic behavior (Figure 2c), and viscoelastic performance (Figure 2d), is of critical importance.

As shown in Figure 2b, all the CMU gel inks exhibited typical shear‐thinning behavior (i.e., the viscosity decreased with the increase in shear rate) owing to the breakage of the network structure under high shear stress (Figure S5b, Supporting Information), which is beneficial for the 3D printable inks because the strong shear‐thinning behavior can enable the smooth extrusion of ink from the nozzle.^[^ ^9^, ^19^ ^]^ Furthermore, the viscosity at a fixed shear rate positively correlates with the ink concentration. For instance, the viscosities at 10 s^−2^ for the 1, 2, 4, and 6 wt.% inks were 7170.97, 11 643.63, 19 980.07, and 27 996.04 Pa s^−1^, respectively (Inset in Figure 2b). This is because both 1D CNFs and MWCNTs with high aspect ratio can form a more robust entangled network at a high solid loading (Figure S6, Supporting Information), leading to higher viscosity. The shear stress as a function of the shear rate (Figure S7a, Supporting Information) can be well fitted by the power–law model (Equation S1 and Table S1, Supporting Information). The calculated consistency coefficient values (*K*) for 1, 2, 4, and 6 wt.% inks were 99.85, 182.74, 285.89, and 387.09, respectively, demonstrating the positive correlation between viscosity and concentration. In addition, the values of the flow behavior index (*n*) are all <1, indicating that the inks possess a shear‐thinning specialty that is especially required for DIW 3D printing.^[^ ^16^ ^]^

Thixotropic capacity was studied by recording the evolution of viscosity over time when alternating low and high shear rates were applied (Figure 2c). During the first 4 min, the CMU gel inks were continuously sheared at an extremely low shear rate of 10^−2^ s^−1^ to simulate the pre‐extrusion process. Subsequently, the shear rate was instantaneously increased to 10^2^ s^−1^ to simulate the printing state of the ink within the nozzle. It was observed that the viscosity rapidly dropped upon changing the shear rate from 10^−2^ to 10^2^ s^−1^ as a result of the disruption of the gel network, which is consistent with the shear‐thinning phenomenon, as shown in Figure 2b. Interestingly, except for the 1 wt.% ink, the viscosity of the other inks could be quickly restored when the shear rate was returned to 10^−2^ s^−1^, indicating that the CNF/MWCNT network of these inks can be rapidly reconstructed upon removal of shear stress. The excellent thixotropic behavior can enable the CMU gel ink to smoothly flow out of the nozzle like a low‐viscosity fluid during printing and simultaneously restore its initial highly viscous gel state after printing, leading to the superior structural stability of the as‐printed constructs.

The viscoelastic performance of the CMU gel inks was further evaluated by recording the storage modulus (*G*′) and loss modulus (*G*″) as functions of the oscillatory stress and angular frequency. As shown in Figure 2d, *G*′ is higher than *G*″ when the oscillatory stress is below the yield stress (i.e., the stress at *G*′ = *G*″), demonstrating the predominantly solid‐like behavior of the inks in this region. Liquid‐like viscoelastic behavior of inks occurs (*G*′ < *G*″) because of the disruption of the gel network structure when the oscillatory stress exceeds the yield stress.^[^ ^20^ ^]^ Notably, the yield stress and *G*′ in the plateau region (i.e., where *G*' is almost independent of oscillatory stress) are proportional to the ink concentration (Figure S8, Supporting Information). In particular, for the 6 wt.% ink, the yield stress (218.89 Pa) and *G*′ in the plateau region (11 345.14 Pa) were much higher than those of the other inks. Moreover, the 6 wt.% ink also exhibited the highest *G*′ value in the angular frequency sweeping measurements (Figure S7b, Supporting Information). The improvement in the *G*′ and yield stress for the 6 wt.% ink is attributed to the increased contact sites between the two 1D building blocks and the formation of a highly entangled network in ink (Figure S6, Supporting Information). It is worth noting that the CMU gel ink with a higher concentration of 7 wt.% was extremely viscous, leading to poor uniformity even after severe homogenization (Figure S9, Supporting Information), which is not desirable for DIW 3D printing. Considering the superior shear‐thinning behavior, outstanding thixotropic capacity, and appropriate viscoelastic performance, the 6 wt.% CMU gel ink was considered the optimal 3D printing ink in this study.

The as‐prepared 6 wt.% ink was then filled into a stainless‐steel printing syringe equipped with a 0.4 mm diameter nozzle to evaluate the extrudability and optimize the printing parameters (i.e., printing pressure and speed). Gradually increasing the printing pressure, the minimum pressure for continuous ink extrusion was confirmed to be ≈45 KPa. Under this condition, a uniform continuous filament can be extruded through a tiny nozzle (Figure S10, Supporting Information). This good extrudability can be explained by the shift in the ink state from gel to fluid within the nozzle, driven by shear stress (Figure 2e). In theory, the shear stress [*τ*(r), Pa] inside the nozzle is independent of the longitudinal distance, whereas it is proportional to the radial distance (r, mm), which can be quantified by the following equation:^[^ ^21^ ^]^

(1) $$\tau \left(r\right)=\frac{\Delta P\cdot r}{2L}$$

where L is the length of the nozzle and ∆*P* is the applied printing pressure. By substituting the actual parameters of L (2 mm) and ∆*P* (45 KPa) into Equation (1), the shear stress as a function of radial distance was plotted (Figure 2f). The shear stress increased linearly from the center to the inner wall of the nozzle (i.e., the r value increased from 0 to 0.2 mm). According to the yield stress (*τ*_y_) of the 6 wt.% ink obtained from the dynamic rheological tests (Figure 2d; Table S2, Supporting Information), the critical radius (*r*_c_) at which the theoretically derived shear stress equals *τ*_y_ (Inset in Figure 2g) was determined to be 0.0195 mm. Note that the inks outside the *r*_c_ suffer from shear stress higher than *τ*_y_, causing this part of the ink to flow like fluid under shearing (Figure 2e). As a result, the fluid state range (i.e., yielding range) of ink inside the nozzle is calculated to be 90.25 vol.% (Table S3, Supporting Information). Such a high‐yielding range indicates that most inks within the nozzle are shear‐thinned, which is responsible for good extrudability, thus allowing further optimization of the printing parameters. To evaluate the effect of printing speed (i.e., substrate moving speed) on filament formation, the filament was extruded on a glass substrate at different printing speeds at an air pressure of 45 KPa (Inset in Figure 2g). Obviously, the width of filament gradually decreases from 1.51 to 0.44 mm as the printing speed increased from 1 to 5 mm s^−1^. However, further increasing the printing speed to 6 mm s^−1^ brings in a severe dragging effect, leading to the discontinuity of the filament (Figure S11, Supporting Information). Since the filament width at a printing speed of 5 mm s^−1^ is close to the printed nozzle diameter (0.4 mm), this speed parameter was considered optimal and applied in the following 3D printing process. As proof of the flexible designability of 3D printing, a variety of 3D architectures with pyramidal, dumbbell, hollow cube, pectinate structure, and CNF font were successfully printed (Figure 2h), all of which possess high geometrical accuracy and shape fidelity without deformation and collapse due to the superior rheology and 3D printability of CMU ink.

More fascinatingly, the DIW 3D printing technique allowed us to construct 3D gel blocks precisely with a tunable layer (Figure 2i, left). To produce N‐doped porous free‐standing architectures for EES applications, the 3D‐printed gel blocks with different layers were further freeze‐dried and annealed to carbonize the CNFs in the blocks and introduce nitrogen of various atomic configurations to the carbon matrix. It should be noted that the annealing temperature was 700 °C, and the in situ N‐doping strategy was achieved by the ammonia gas generated from the decomposition of urea in the block during the annealing process.^[^ ^13a^ ^]^ Interestingly, the post‐treatment of freeze‐drying and annealing did not alter the shape fidelity of the 3D printed blocks (Figure 2i), which is probably attributed to the high solid loading of CMU ink (6 wt.%) and the high proportion of MWCNTs (70 wt.%) in the CMU ink, which can suppress the structural shrinkage during post‐treatment. The actual heights of the post‐treated blocks with 3, 4, 5, and 6 deposited layers were 1.22, 1.61, 2.03, and 2.43 mm, respectively (Figure S12 and Table S4, Supporting Information), which are close to the theoretical heights (i.e., the heights of the designed model), further demonstrating the superior shape fidelity of the 3D‐printed blocks.

**Figure 3:**
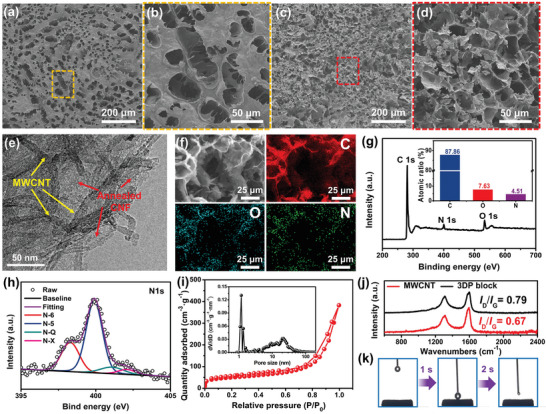
Morphology and chemical structure of the 3D‐printed, freeze‐dried, and annealed block: a) surface and c) cross‐sectional view SEM images. b,d) The magnified SEM images correspond to the yellow and red dashed frame parts in (a) and (c), respectively. e) TEM image of the block. f) EDS mapping of C, O, and N elements inside the block. g) XPS spectra of the block. (h) High‐resolution N 1s spectra. i) N_2_ adsorption and desorption isotherms, inset is the obtained pore size distribution using the DFT method. j) Raman spectroscopy of the MWCNT and block. k) Contact angle test on the block.

The post‐treated block was comprehensively characterized using a wide spectrum of analytical techniques, including scanning electron microscopy (SEM), energy‐dispersive spectroscopy (EDS) mapping, transmission electron microscopy (TEM), N_2_ adsorption and desorption isotherms, X‐ray photoelectron spectroscopy (XPS), Raman spectroscopy, and contact angle tests. **Figure** **3**a,b, and Figure S13 (Supporting Information) present the surface‐view SEM images of the post‐treated block, from which macropores with diameters ranging from 10 to 100 µm were identified. In addition, the cross‐sectional SEM images show that the pores were interconnected to form a porous network (Figure 3c,d; Figure S14, Supporting Information). These macropores that appeared both on the surface and inside the block were conducive to the electrochemical performance because they could facilitate the penetration of the electrolyte and increase the electrolyte‐accessible surface area.^[^ ^22^ ^]^ The highly magnified TEM image of the block shows that the annealed CNFs wrapped around the MWCNTs in an amorphous carbon state after post‐treatment (Figure 3e; Figure S15, Supporting Information). This implies that the annealing treatment can convert the pristine entangled network into an encapsulated continuous conductive network, thereby endowing the block with excellent electronic conductivity (Figure S16, Supporting Information). Furthermore, the EDS mapping images of the inside morphology display a uniform distribution of doped N, whereas the detected O implies the existence of oxygen‐containing groups (Figure 3f; Figure S17, Supporting Information). Consistent with the EDS mapping results, the C 1s, O 1s, and N 1s peaks were detected in the XPS survey spectra (Figure 3g), and the corresponding atomic ratios were calculated as 87.86%, 7.63%, and 4.51%, respectively. The high‐resolution N 1s spectrum (Figure 3h) can be deconvoluted into four individual peaks: N–6 (398.3 eV), N–5 (399.9 eV), N–Q (401.1 eV), and N–X (402.1 eV), confirming that the N atom was successfully doped into the block. Note that the presence of N–Q can enhance the conductivity of the block by promoting the transfer of electrons, while the N–6, N–5, and N–X can act as redox centers to contribute to the pseudo‐capacitance. Therefore, introducing N atoms is anticipated to endow the block with superior electrochemical performance when used as an electrode for a supercapacitor.^[^ ^13a^ ^]^ The high‐resolution O 1s spectrum and its deconvoluted peaks of C=O (530.6 eV), C—O (532.2 eV), and O—C=O (534.3 eV) (Figure S18, Supporting Information) revealed the existence of oxygen‐containing groups on the block surface.^[^ ^23^ ^]^ In addition to the micropores and surface electrochemical activity, the block possesses a hierarchical pore structure, as revealed by the N_2_ adsorption and desorption isotherms. As shown in Figure 3i, a strong adsorption volume at low pressures and hysteresis in the intermediate pressure range were observed, indicating the existence of a hierarchical porous structure in the block.^[^ ^24^ ^]^ Based on Brunauer–Emmett–Teller (BET) model, the specific surface area of the block was calculated to be 171.76 m^2^ g^−1^. The pore size distribution obtained by the Density Functional Theory (DFT) method shows that the peak of the micropores is located at 1.17 nm, whereas the mesopores are widely distributed in the range of 5–40 nm (Inset in Figure 3i). Such hierarchical nanopores inside a block are expected to improve the transport efficiency of electrolyte ions and provide sufficient active sites for the accumulation of charges or ions.^[^ ^25^ ^]^ The Raman spectra of the block and MWCNTs are shown in Figure 3j, and two well‐known G‐band peak (≈1590 cm^−1^) and D‐band peak (≈1320 cm^−1^) related to the graphitic structure and structural defects were observed. In addition, the structural defect degree of the printed block was higher than that of the MWCNT building block, as demonstrated by the higher peak intensity ratio of the D band to the G band (*I*_D_/*I*_G_ = 0.79) for the printed block.^[^ ^26^ ^]^ Thanks to the synergistic effects of the hierarchical porous structure, doped N atoms and oxygen‐containing groups on the surface, and a high degree of structural defects, the block exhibited superb wettability with rapid water infiltration within 2 s (Figure 3k).

Mechanical performance is non‐negligible for 3D‐printed porous materials because the well mechanical strength of 3D‐printed materials can resist some degree of external force in practical use without damage. To evaluate the mechanical properties of our 3D‐printed carbon block, a cyclic compressive test was conducted, and the stress–strain curves of the compressing–releasing cycles are shown in Figure S19a (Supporting Information). The compressive strength of the 6‐layer block reaches 101.7 KPa at a compressive strain of 30% in the first cycle, which was higher than that of the 3D‐printed CNF/SWCNT carbon lattice structure,^[^ ^13a^ ^]^ 3D‐printed carbon aerogel,^[^ ^27^ ^]^ and 3D MXene/rGO aerogel.^[^ ^28^ ^]^ However, the compression recovery rate was 28.83%, indicating that the compression recovery performance was not good. Fortunately, the compression strength of the 3D‐printed block at a strain of 30% in the 2nd, 5th, and 10th cycles were determined to be 97.2, 94.1, and 92.3 KPa, demonstrating that mechanical strength can be maintained well in cyclic compression (Figure S19b, Supporting Information). Specifically, the recovery rates referenced to the 1st cycle are registered at 89.71%, 85.32%, and 81.16% after 2, 5 and 10 compression cycles, respectively. Overall, these results demonstrate the good mechanical strength of our 3D‐printed block, but the compression recovery performance needs to be improved in the future. It should be mentioned that the poor compression recovery performance is because the annealing treatment at 700 °C transformed the robust CNF network into rigid carbon in the printed block, as confirmed by the TEM images (Figure 3e).

**Figure 4:**
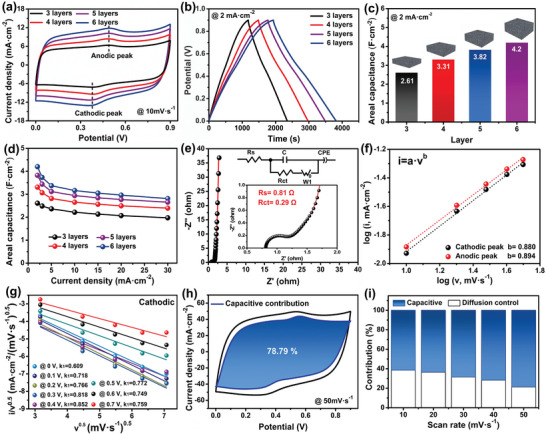
Electrochemical performance of the 3D‐printed carbon block: a) CV profiles (10 mV s^−1^) and b) GCD curves (2 mA cm^−2^) of the block with different layers. c) The areal capacitance of the block with different layers at 2 mA cm^−2^. d) The areal capacitance of the block with different layers at various current densities (2–30 mA cm^−2^). e) Nyquist plots and insets are the enlarged plots at the high‐frequency range (bottom) and the equivalent circuit model (top). f) Linear fitting plot of the exponential value of peak current versus the exponential value of scanning rate. g) Linear relationship between the cathodic current response to the square root of the scan rate and the square root of the scan rate at a given potential. h) CV profiles and the corresponding capacitive contribution plots at 50 mV s^−1^. i) Capacitive‐ and diffusion control‐contribution at different scan rates.

With the advantages of tailorable thickness, hierarchical porous nanostructure (macro‐, meso‐, and micropores), high electronic conductivity (10.91–12.39 S m^−1^, Figure S16b, Supporting Information), appropriate N heteroatom doping level (4.51%), and good mechanical strength, the 3D‐printed carbon blocks hold a great promise as electrodes in the EES applications. To evaluate the influence of thickness on the energy storage capacity, the electrochemical performance of the blocks with different layers (the height, mass loading, and area are summarized in Table S4, Supporting Information) was tested in a typical three‐electrode configuration with 1 m H_2_SO_4_ as the electrolyte. The cyclic voltammetry (CV) profiles (Figure S20, Supporting Information) of the blocks at 10 mV s^−1^ displayed an approximately rectangular shape with a pair of broad redox peaks, which were probably contributed by the reaction of H^+^ ions in the acid electrolyte with oxygen‐containing groups on the carbon block surface (C=O + H^+^ + e^−^ ↔ C—OH & COO^−^ + H^+^ + e^−^ ↔ COOH).^[^ ^8^, ^29^ ^]^ Moreover, the increase in the area of the CV profiles (**Figure** **4**a), as well as the discharging time in galvanostatic charge–discharge (GCD) curves (Figure 4b) with an increase in the number of printed layers confirms the efficient utilization of the active materials. These promising results also indicate that the number of deposited layers can effectively control the electrochemical performance of the block, as evidenced by the improvement in the areal capacitance as the number of printed layers increases (Figure 4c). To quantitatively evaluate the rate capability of the block, GCD measurements at different current densities in the range of 2–30 mA cm^−2^ were performed (Figure S21, Supporting Information), and the obtained areal capacitance is shown in Figure 4d. The areal capacitance of the blocks with 3, 4, 5, and 6 layers reach 2.61, 3.31, 3.82, and 4.2 F cm^−2^ at 2 mA cm^−2^, respectively, which can be maintained to 1.97, 2.39, 2.65, and 2.81 F cm^−2^, even when the current density increases by 15‐fold (30 mA cm^−2^). The desirable areal capacitance and superior rate capability of the 3D‐printed carbon block are the synergistic results of 1) hierarchical pores combined with high specific surface area, 2) N‐doped carbon structure, and 3) high mass loading (15.2–30.2 mg cm^−2^) controlled by 3D printing. Particularly, for the N‐dopping contribution, it was widely reported that doping the N atom enhanced electronic conductivity, wettability, and structural defects for the carbon‐based electrode materials, which is beneficial for the improvement of electrochemical performance.^[^ ^30^ ^]^ For comparison, the CNF/MWCNT (CM) block was also printed using the CNF/MWCNT ink under the same protocol as the CMU block, followed by freeze‐drying and annealing treatments. Although the CM block exhibits good capacitive performance, its areal capacitance (2.92 F cm^−2^ at 2 mA cm^−2^) is inferior to the CMU block and has lower retention of 55% at the high current density of 30 mA cm^−2^ (Figure S22, Supporting Information). The contrasting results reveal the effectiveness of N doping in improving the capacitive and rate performance. On the other hand, the well‐connected hierarchical nanoporous structure consisting of macro‐, meso‐, and micropores in the block also has positive effects on the electrochemical performance, as it can improve electrolyte accessibility and facilitate the diffusion efficiency of electrolyte ions. More importantly, the 3D printing technique allows precise control of the mass loading of the active materials via tuning the thickness of the electrode, leading to a high areal capacitance. The areal capacitance of the block (6‐layer, 2.43 mm thick, 4.2 F cm^−2^) was higher than that of some thick electrodes (Figure S23, Supporting Information), such as surface‐functionalized 3D‐printed graphene aerogel (4 mm in thickness, 3.231 F cm^−2^),^[^ ^8a^ ^]^ N, P‐doped carbon wood (2.4 mm in thickness, 2.98 F cm^−2^),^[^ ^31^ ^]^ and 3D‐printed porous graphene aerogel (1 mm in thickness, 0.207 F cm^−2^).^[^ ^12^ ^]^ It is noted that the areal capacitance is less than the 3D‐printed CNF/SWCNT thick electrode and the 3D‐printed AC/CNT/rGO electrode,^[^ ^13^, ^32^ ^]^ which is because of the N‐doping level or specific surface area is not superior to that of these two electrodes. In addition to the areal capacitance, our printed thick block with six layers delivered a good specific capacitance of 139 F g^−1^ and volumetric capacitance of 17.3 F cm^−3^ at 2 mA cm^−2^ (Figure S24, Supporting Information).

To gain better insight into the capacitive behavior of the 3D‐printed N‐doped carbon block, electrochemical impedance spectroscopy (EIS) and electrochemical kinetics analysis were performed on the six‐layer block (Figure 4e–i). The Nyquist plot in Figure 4e displays a small semicircle in the high‐frequency region and a nearly vertical line in the low‐frequency region, suggesting excellent conductivity and good capacitive behavior of the block. An equivalent circuit model was utilized to quantify the charge transfer resistance (*R*_ct_, diameter of the semicircle) and equivalent series resistance (*R*_s_, x‐intercept) from the EIS curve (Inset in Figure 4e). The *R*_s_ and *R*_ct_ are 0.81 and 0.29 Ω, respectively, revealing that the block electrode has low internal resistance and outstanding electron transfer capacity, which is attributed to the continuous carbon‐based conductive network and N‐doped structure inside the block. A kinetic analysis was further conducted to determine the charge storage mechanism of the electrode. The relationship between the peak current density (i) and scan rate (v) in the CV profile follows the power‐law model (i.e., i = av^b^, where b = 1 represents the surface‐controlled capacitive storage process, while b = 0.5 indicates the diffusion‐controlled process).^[^ ^33^ ^]^ By linearly fitting the logarithm of the peak current density against the logarithm of the scan rate, the b values corresponding to the cathodic and anodic peaks were calculated to be 0.880 and 0.894, respectively (Figure 4f), suggesting that both capacitive‐ and diffusion‐controlled behaviors coexist in the charge storage process. The contribution ratio of the above two portions in the CV profile at a given scan rate can be separated as i = k_1_v+k_2_v^0.5^, where k_1_v and k_2_v^0.5^ reflect the capacitive‐ and diffusion‐controlled contributions, respectively. In other words, by linearly fitting i/v^0.5^ as a function of v^0.5^, a series of k_1_ values at a given potential can be obtained to determine the capacitive contribution ratio (Figure 4g; Figure S25, Supporting Information).^[^ ^34^ ^]^ It was found that the capacitive‐controlled contribution ratio increased from 61.45% to 78.79% when the scan rate gradually increased from 10 to 50 mV s^−1^ (Figure 4h,i), demonstrating the favorable capacitive behavior and good charge kinetics of the 3D‐printed thick carbon block.

Encouraged by the superior electrochemical properties of 3D‐printed carbon block, a symmetrical supercapacitor (SC) system was assembled using two six‐layer blocks as free‐standing electrodes, together with 1 m H_2_SO_4_ as the electrolyte (Figure S26, Supporting Information). The CV profiles of the SC preserve a rectangular shape in the scan rate range of 10–100 mV s^−1^ (Figure S27a, Supporting Information), suggesting ideal capacitive behavior and good rate capability. Based on the GCD curves, the areal capacitance of the SC were obtained (Figure S27b,c, Supporting Information). The SC device delivers an areal capacitance of 2.89 F cm^−2^ at 2 mA cm^−2^ and 1.73 F cm^−2^ at a high current density of 30 mA cm^−2^, surpassing some of the previously reported SCs, such as 3D‐printed rGO (0.639 F cm^−2^ at 4 mA cm^−2^),^[^ ^35^ ^]^ 3D‐printed CMC/rGO (1.57 F cm^−2^ at 2 mV s^−1^),^[^ ^36^ ^]^ vertical‐section wood/MXene (0.805 F cm^−2^ at 0.5 mA cm^−2^),^[^ ^37^ ^]^ and PVA‐PANi (0.3448 F cm^−2^ at 0.3 mA cm^−2^).^[^ ^38^ ^]^ Furthermore, the SC possesses a high capacitance retention of 92% after 10 000 charge–discharge cycles at 30 mA cm^−2^ (Figure S27d, Supporting Information), showing a remarkable cycling life. Overall, the impressive electrochemical performance of our 3D‐printed carbon block in three‐ and two‐electrode systems confirms its promising application prospects as electrodes in EES devices.

**Figure 5:**
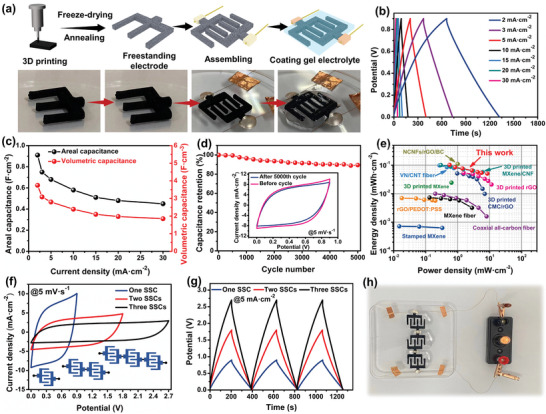
Electrochemical performance of the 3D‐printed quasi‐solid‐state symmetric supercapacitor (QSSC): a) Schematic diagram and digital images of the fabrication process for the QSSC device. b) GCD curves at different current densities (2–30 mA cm^−2^). c) Areal‐ and volumetric‐capacitance of the QSSC at various current densities. d) Capacitance retention measured for 5000 cycles at a current density of 30 mA cm^−2^, inset is the CV profiles at 5 mV s^−1^ before and after 5000 cycles. e) Areal Ragone plot of 3D‐printed QSSC device with another reported device. f) CV profiles at 5 mV s^−1^ and g) GCD curves at 5 mA cm^−2^ for the tandem devices with different numbers of QSSC. h) The tandem device with three QSSC can power a small ball bulb.

Finally, to demonstrate the advantages of the flexibility and customization of the 3D printing technique, two interdigitated N‐doped carbon architectures with six layers were fabricated using the 3D printing technique, followed by freeze‐drying and annealing treatments (**Figure** **5**a; Figure S28, Supporting Information). Subsequently, a quasi‐solid‐state symmetric supercapacitor (QSSC) was assembled using two interdigitated N‐doped carbon architectures as electrodes and a PVA/H_2_SO_4_ gel as the electrolyte. The as‐prepared QSSC device showed good capacitive characteristics and reversible charge/discharge behavior, as reflected by the GCD curves at various current densities based on the two‐electrode areas (2.304 cm^−2^) (Figure 5b). The QSSC device delivered an areal capacitance of 1.47 F cm^−2^ at 2 mA cm^−2^ and 0.72 F cm^−2^ at 30 mA cm^−2^ based on the area of the two electrodes (Figure S29, Supporting Information). To better reflect the actual performance of a complete device, the charge/discharge current in the GCD measurement was still based on the area of the two interdigitated electrodes, while the device area of the two electrodes and their gap (3.704 cm^−2^, Figure S28, Supporting Information) were applied to Equation S5 (Supporting Information) to evaluate the areal capacitance. Accordingly, the QSSC device could deliver an areal capacitance of 0.91 F cm^−2^ (3.74 F cm^−3^) at 2 mA cm^−2^ and 0.45 F cm^−2^ (1.85 F cm^−3^) at 30 mA cm^−2^ (Figure 5c), which surpass those of the previously reported state‐of‐art devices (Table S5, Supporting Information), such as 3D‐printed carbon aerogel (0.231 F cm^−2^ at 1.2 mA cm^−2^), 3D‐printed rGO (0.639 F cm^−2^ at 4 mA cm^−2^), carbon wood (0.846 F cm^−2^ at 1 mA cm^−2^), and PANi/rGO/Fabric (0.564 F cm^−2^ at 1 mA cm^−2^). Moreover, after 5000 charge–discharge cycles at 30 mA cm^−2^, the QSSC has a capacitance retention of 89% (Figure 5d), and it still possesses a good capacitive behavior, as confirmed by the CV profiles (inset in Figure 5d). The Ragone plot shows that the QSSC achieves high areal energy densities from 0.10 to 0.05 mWh cm^−2^ at areal power densities from 0.56 to 8.44 mW cm^−2^, which are comparable to other state‐of‐art symmetric supercapacitors reported previously (Figure 5e; Table S6, Supporting Information).^[^ ^16^, ^20^, ^35^, ^36^, ^39^ ^]^ On the other hand, the volumetric energy density of QSSC reaches 0.42 mWh cm^−3^ at a power density of 2.30 mW cm^−3^, and 0.21 mWh cm^−3^ at a high power density of 34.72 mW cm^−3^, which is comparable to other reported symmetric device (Figure S30, Supporting information). To demonstrate the practical use of QSSCs, we assembled tandem‐connected circuits of the QSSCs. Compared to the single QSSC, the potential window of the tandem devices with two and three QSSCs was remarkably enhanced to 1.8 and 2.7 V, respectively (Figure 5f,g). Owing to the high energy output resulting from the wide potential window (E = 1/2CV^2^, where *E* is the energy density, *C* is the capacitance, and *V* is the potential window), three printed QSSCs in series connection can power a small ball bulb (Figure 5h), showing excellent integrability and practicability.

## 3 Conclusion

In summary, a 3D printable CMU ink consisting of CNFs, MWCNTs, and urea was successfully developed by studying the concentration‐dependent rheological performance of CMU inks and optimizing the 3D printing parameters. The CMU ink with a solid concentration of 6 wt.% exhibited excellent rheological behavior (i.e., profound shear shinning peculiarity, superior thixotropy, and desirable viscoelasticity) and 3D printability (i.e., extrudability, geometrical accuracy, and shape fidelity). The free‐standing hierarchical porous carbon blocks for high‐performance supercapacitors were realized by freeze‐drying and annealing. The well‐designed hierarchical porous structure, high electrical conductivity, and superb wettability induced by nitrogen doping with different atomic configurations enabled fast electron transport and ion diffusion within the printed block, resulting in excellent electrochemical performance. As proof of the structural designability of the 3D printing technique, an interdigitated QSSC device was successfully manufactured, which exhibited a high areal capacitance of 0.91 F cm^−2^ at 2 mA cm^−2^, good cycling stability with capacitance retention of 89% after 5000 cycles, and an energy density of 0.10 mWh cm^−2^ at 0.56 mW cm^−2^. We believe this work provides detailed guidance for optimizing the printability of ink used for the 3D printing of high‐performance supercapacitors.

## Conflict of Interest

The authors declare no conflict of interest.

## Supporting information

Supporting Information

## Data Availability

The data that support the findings of this study are available from the corresponding author upon reasonable request.
