# Supplementary material for: 3D Printed Nitrogen‐Doped Thick Carbon Architectures for Supercapacitor: Ink Rheology and Electrochemical Performance
Source: Adv Sci (Weinh). 2023 Feb 7;10(10):2206320. doi: 10.1002/advs.202206320 (PMC10074055; doi:10.1002/advs.202206320)
Supplement: Supplementary file 1 — Supporting Information [file ADVS-10-2206320-s001.pdf]

Supporting Information

3D Printed Nitrogen-Doped Thick Carbon Architectures for Supercapacitor: Ink Rheology and Electrochemical Performance

Guoqiang Zhou[1], Mei-Chun Li[1,2,], Chaozheng Liu[1], Chuhang Liu[1], Zhenglin Li[1], and Changtong Mei[1,]**

Experimental Section

***Preparation of Cellulose Nanofibers (CNFs) From Oil Palm Wood Trunk*:**

First, the oil palm wood trunk wood was crushed into fibers using a plant crusher. The oil palm fibers were treated with hot-water, $NaClO_{2,}$ and NaOH to remove the impurity, lignin, and hemicellulose according to our previously reported work.[1] Next, the obtained white oil palm cellulose was treated by a TEMPO-mediated oxidation process. In brief, 5 g oil palm cellulose, 0.08 g 2,2,6,6- tetramethylpiperidine-1-oxyl (TEMPO), and 0.5 g NaBr were dispersed into 200 mL deionized (DI) water with stirring for 10 min to form the homogeneous slurry. Then, 28.8 g NaClO solution (available chlorine of 6 %) was dropwise added into the slurry with stirring. Subsequently, the pH of the hybrid suspension was adjusted to 10 using 0.5 M NaOH and HCl solutions to initiate the TEMPO-mediated oxidation reaction. Note that the reaction can oxidize the hydroxy groups (-OH) at the C6 position of cellulose into the negatively charged carboxyl groups (COO^-). When the pH was constant, the TEMPO-oxidized oil palm cellulose was washed and centrifuged using DI water several times until the pH of that became neutral. The obtained samples were dispersed in 200 mL DI water and ultrasonicated at 600 W for 30 min to produce the cellulose nanofibers (CNFs). Finally, the suspension was centrifuged at 5000 rpm for 30 min to remove non-nanofibrillated cellulose fibers. The supernatant containing the CNFs was carefully collected for later use.

Dispersion of Multiwalled Carbon Nanotubes (MWCNTs) in CNF Suspension:

The CNF suspension was diluted to a concentration of 0.2 wt%. Then, MWCNTs (MWCNTs:CNFs=7:3) were added into the CNF suspension with stirring for 30 min. The hybrid suspension was ultrasonicated at 300 W for 1 h to enhance the dispersion of MWCNTs in CNF suspension.

Formulation of the CNF/MWCNT/Urea (CMU) Ink:

The well dispersed CNF/MWCNT (CM) suspension was concentrated to remove part of the water using the highly hydrophilic absorbent beads according to previous studies.[2] In brief, the absorbent beads were added to the CM suspension, and the suspension containing the beads was artificially stirred slowly with a glass rod every 5 minutes to ensure homogeneity. After 30 min, the highly concentrated suspension was carefully collected and homogenized by a vacuum planetary high-speed mixer to remove air bubbles. This process was repeated several times until the concentration of CM gel was 4 wt%. Subsequently, the urea was dispersed into the CM gel to achieve the CNF/MWCNT/Urea (CMU) gel by homogenizing. The weight ratio of MWCNT to urea in the gel was fixed at 1:5. To obtain the gel inks with different concentrations (1, 2, 4, and 6 wt%), the CMU gel was further concentrated using the absorbent beads as mentioned above, or diluted with deionized water and homogenized by the vacuum planetary high-speed mixer.

Rheology Measurement :

Before 3D printing, the rheological properties of the gel inks were tested using a MARS60 rheometer (Thermo Fisher Scientific Inc. Germany) equipped with a parallel plate geometry (diameter 35 mm) to evaluate the 3D printability. All experiments were carried out at 25 °C. The steady shearing measurements were performed to record the viscosity at the

shear rates ranging from 10^{-2} to 10^{3} s^{-1}. The shear stress as a function of shear rate could be obtained using the equation S1:

$$\tau = \gamma \cdot \eta \qquad \text{(S1)}$$

where the τ is the shear stress (Pa), γ and η are the shear rate (s^{-1}) and viscosity (Pa·s), respectively. The τ as a function of γ can be further fitted by equation S2 to obtain the consistency coefficient (K) and flow behavior index (n).

$$\tau = K \cdot \gamma^{n} \qquad \text{(S2)}$$

For the dynamic rheological tests, the storage modulus and loss modulus as a function of oscillatory stress were recorded at a fixed frequency of 1Hz, while the angular frequency sweep measurements were carried out from 1 to 10^{2} rad·s^{-1}. The thixotropic behavior was evaluated by recording the change in viscosity over time when applying the alternating low (10^{-2} s^{-1}) and high shear rates (10^{2} s^{-1}).

3D Printing of Gel Ink and Post-treatment :

The 6 wt% CMU gel ink with exceptional rheological properties was chosen for 3D printing of gel architectures using a Dr. INVIVO 4D bioprinter (ROKIT Healthcare, INC, South Korea) equipped with a pneumatic dispenser. The gel ink was loaded into a printing syringe, which was then centrifuged at 2000 rpm for 5 min. To evaluate the filament formation and optimize the printing speed, the filament was extruded through the printed nozzle (0.4 mm in diameter) on the glass substrate under different printing speeds (i.e., substrate moving speed = 1, 2, 3, 4, and 5 mm·s^{-1}) at the printing pressure of 45 KPa. The printing speed is optimized when the width of extruded filament is close to the nozzle diameter. The models were designed using a 3D MAX 2019 software and then translated to G-code instruction for printing by a Newcreator software associated with the printer. The gel architecture was printed layer-by-layer on a glass substrate by executing the G-code

instruction using the optimal printing speed. The printed gel structures were frozen at -4 ℃ and then freeze-dried for 48 h to produce the freestanding 3D architectures. Subsequently, the 3D architectures were annealed at 700 ℃ (5 ℃·min^{-1}) under the N_2 atmosphere for 2 h to achieve the effect of N-dopping and carbonize the CNFs. For comparison, the 6 wt% CM gel ink without urea was used to print the gel block, which was then transformed into the N-undoped carbon block after freeze-drying and annealing treatments.

Material Characterization:

Zeta potential values of the CNF and CM suspension were measured by a ZetaTrac analyzer (Microtrac, Largo, USA). The morphologies of CM were observed using a transmission electron microscope (TEM, JEM-1400, JEOL) with an accelerating voltage of 80 kV. The width of the extruded filaments and the height of the post-treated blocks were measured using the Image J software according to the actual ruler. The morphology and energy-dispersive spectroscopy (EDS) mapping of the post-treated printed block were characterized using a filed-emission scanning microscope (FE-SEM, JSM-7600F, JEOL) operated at 15 kV. A high-resolution transmission electron microscope (HRTEM, JEM-2100, JEOL) with an accelerating voltage of 200 kV was applied to obtain the morphology of the post-treated block. The N_2 adsorption/desorption isotherms were measured by a Micrometritics ASAP 2020 analyzer. The specific surface area and pore size distribution of the post-treated block were obtained using the Brunauer–Emmett–Teller (BET) method and the density functional theory (DFT), respectively. The atomic configurations of the block were characterized by an X-ray photoelectron spectrometer (XPS, AXIS UltraDLD, Shimadzu) at 225 W with an Al Kα X-ray source. Raman spectroscopy was recorded by a laser Raman spectrometer (DXR532, Themor, USA). The wettability of the block was assessed by contact angle testing equipment (DSA100S, German Dataphysics). The

compressive tests for the block (6-layer) was conducted using a Sansi Zongheng UTM5000 electronic universal testing machine with a loading rate of 1 mm·min^{-1} at room temperature.

The linear sweep voltammetry was performed using a CHI 760E electrochemical workstation (Chenhua, Shanghai, China) to calculate the electronic conductivity of the blocks. Before testing, two copper wires were fixed at both sides of the block using the silver plate, as shown in the inset of Figure S16a. According to the Ohm law, the resistance (R, Ω) of the block was determined using the equation S3:

$$R=U/I \quad \text{(S3)}$$

where U and I are the voltage (V) and current (A), respectively. Then, the conductivity (C, S·m^{-1}) was calculated by equation 4.

$$C=L/(R{\cdot}b{\cdot}d) \quad \text{(S4)}$$

where L, b, and d are the length, width, and height of the block, respectively.

Fabrication of 3D Printed N-dopped Carbon-based Symmetric Supercapacitors:

For the printed block-based symmetric supercapacitor, two blocks (6 layers) were fixed by platinum electrode clips and directly used as working electrodes in 1 M H_2SO_4 electrolyte. The image of the two-electrode configuration is shown in **Figure S26**.

The quasi-solid-state symmetric supercapacitor (QSSC) was constructed using two interdigitated N-doped carbon electrodes (6 layers) fabricated by 3D printing, freeze-drying, and annealing treatments. The designed model and its dimension are displayed in **Figure S28**. Then, two interdigitated electrodes were assembled onto the glass substrate, and the copper wires were fixed on both sides of the electrode using the silver paste. After the silver curing at the room termperature for 24h, the interdigitated electrodes and the copper wires can been fixed and well adhere to the glass substrate. PVA/H_2SO_4 gel electrolyte was prepared as following. In the beginning, about 1 g PVA powder was added into 10 mL DI water under

continuous stirring at 85 °C until the PVA was dissolved completely. After cooling to room temperature, about 1 mL H_2SO_4 was dropwise added into the mixture and stirred for 1h. The electrolyte was continuously coated on the interdigitated electrodes and the device was placed in the air for 24 h to evaporate the water from the electrolyte.

Electrochemical Measurement:

The electrochemical performance of block and symmetric supercapacitor devices was investigated using a CHI 760E electrochemical workstation (Chenhua, Shanghai, China). The height, area, and mass loading of the printed block with different layers are shown in **Table S4**. The printed blocks with different layers were tested in a three-electrode configuration with 1 M H_2SO_4 electrolyte, in which the block, mercury/mercurous sulfate electrode, and platinum sheet electrode were applied as working electrode, reference electrode, and counter electrode, respectively. For the symmetric supercapacitor device, two blocks with 6 layers (geometric area: 1 cm^2; height: ~2.43 mm; mass loading: ~30.2 $mg \cdot cm^{-2}$) were used as working electrodes in 1 M H_2SO_4 electrolyte, and the electrochemical performance of the device was measured by applying a two-electrode configuration (Figure S26). In addition, the performance of the quasi-solid-state symmetric supercapacitor device was also tested by a two-electrode configuration that connected with the two copper wires. The geometric area and height of the one designed interdigitated electrode are 1.152 cm^{-2} and ~2.43 mm (6 layers), respectively. The mass loading of the interdigitated electrode is ~29.5 $mg \cdot cm^{-2}$.

Cyclic voltammetry (CV), Galvanostatic charge-discharge (GCD) electrochemical impedance spectroscopy (EIS) were measured to evaluate the electrochemical performance. The EIS test was carried out at an open circuit potential in the frequency range of 10^{-2} to 10^3 kHz with a voltage amplitude of 10 mV. The long-term cycling stability was evaluated by repeating the GCD measurement at 30 $mA \cdot cm^{-2}$. The capacitance, energy density, and power density were calculated based on the discharge curves in GCD measurements. Specifically,

the areal capacitance (C_s, F·cm^{-2}), specific capacitance (C_g, F·g^{-1}), and volumetric capacitance (C_v, F·cm^{-3}) were calculated using the following equations:

$$C_s=(I\cdot t)\cdot(\Delta U\cdot s)^{-1} \quad \text{(S5)}$$

$$C_g=(I\cdot t)\cdot(\Delta U\cdot m)^{-1} \quad \text{(S6)}$$

$$C_v=(I\cdot t)\cdot(\Delta U\cdot V)^{-1} \quad \text{(S7)}$$

where the I (A) is the charge-discharge current, t (s) is the discharge time, ΔU (V) is the voltage change in the discharge process. The s, m, and V stand for the area (cm^{-2}), mass (g), and volume (cm^{-3}) of the electrode, respectively. Note: when the measurements were conducted in three electrode system, the appiled current density was based on the area of one electrode (1 cm^{-2}), and the m was the mass of one electrode; When the tests were carried out in two electrode system with 1M H_2SO_4, the appiled current density was based on the area of two electrodes, and the area (s) was the area of two electrodes (2 cm^{-2}); For the QSSC device, the appiled current density was based on the area of two interdigitated electrodes (Figure S28, 2.304 cm^{-2}), while the area (s) in equation S5 was setted as the area of two interdigitated electrodes and their gap (Figure S28, 3.704 cm^{-2}).

The energy density (E, Wh·kg^{-1}, mWh·cm^{-2}, mWh·cm^{-3}) and power density (P, W·kg^{-1}, mW·cm^{-2}, mW·cm^{-3}) were determined according to following equations:

$$E=(1/2)\cdot C\cdot(\Delta U)^2 \quad \text{(S8)}$$

$$P=3600\cdot E\cdot t^{-1} \quad \text{(S9)}$$

where C is capacitance (F·g^{-1}, mF·cm^{-2}, F·cm^{-3}) obtained from equations S5, S6, ΔU (V) and t (s) are voltage change and discharge time in the discharge process, respectively.

Figures

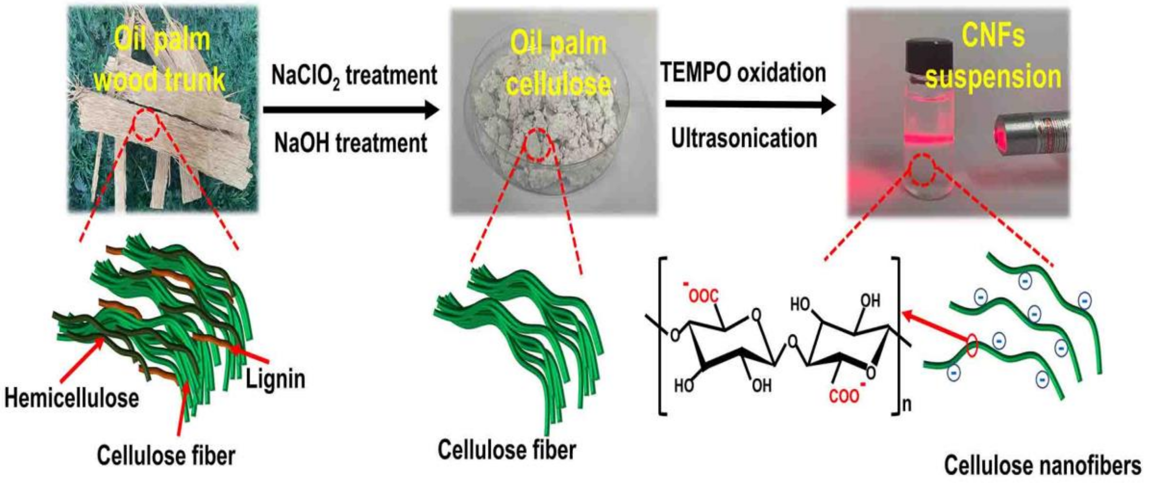

Figure S1. The preparation process of CNFs from the oil palm wood trunk.

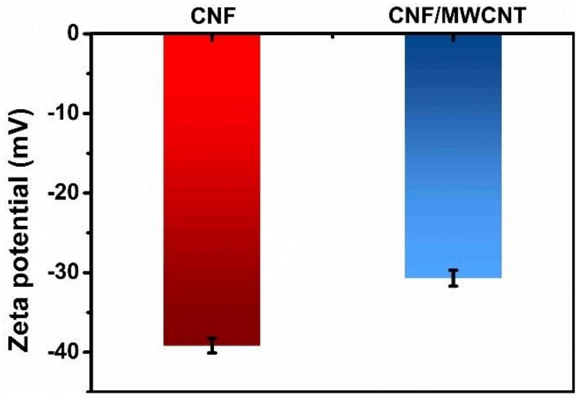

Figure S2. The Zeta potential values of CNF and CNF/MWCNT suspension. Note the concentration of CNF and CNF/MWCNT suspensions is 0.2 wt% and 0.66 wt%, respectively.

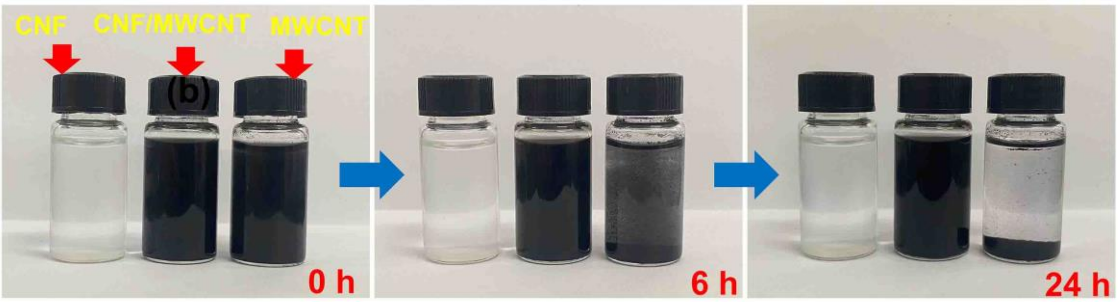

Figure S3. Digital images of CNF, CNF/MWCNT, and MWCNT suspensions at different standing times.

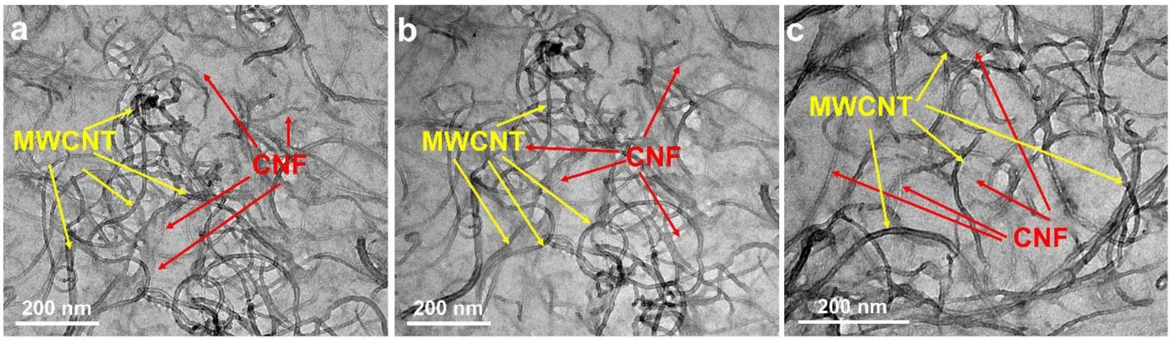

Figure S4. TEM images of CNF/MWCNT hybrid.

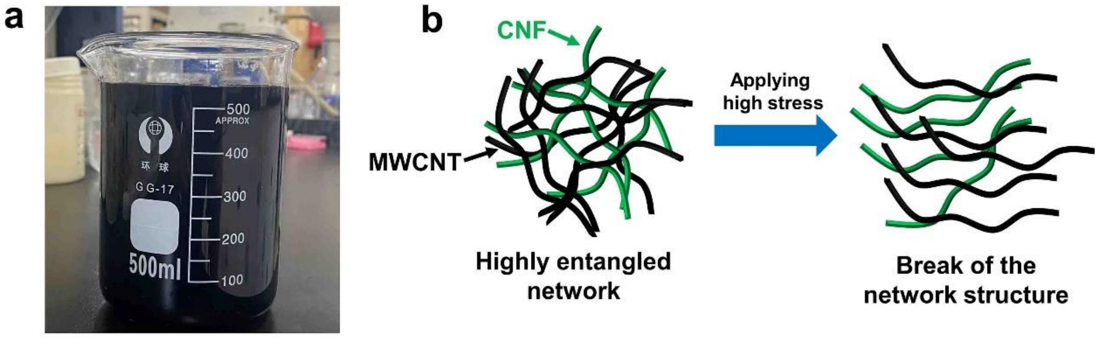

Figure S5. a) Digital image of more than 500 mL of well-dispersed CNF/MWCNT suspension prepared by the dispersing, and ultrasonication approach. b) Schematic illustration of the proposed destruction of the CNF/MWCNT network under high shear stress.

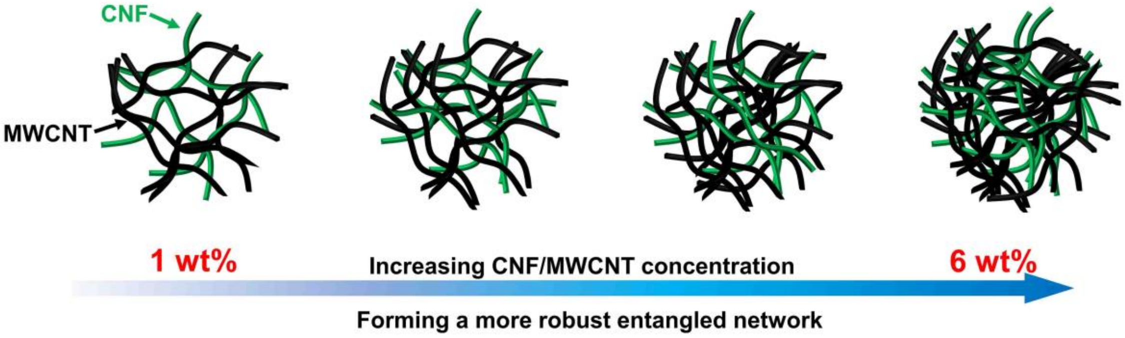

Figure S6. Schematic illustration of the formation of a more robust entanglement network with the increase of CNF/MWCNT concentration.

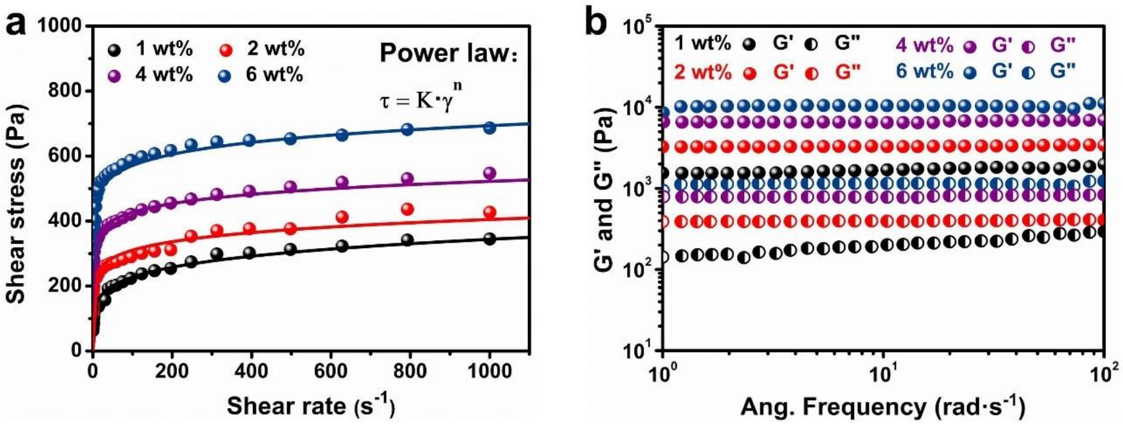

Figure S7. a) Shear stress as a function of shear rate of CMU gel ink with different concentrations, which can be fitted by the Power law model. b) G′ and G″ as a function of the angular frequency of CMU gel ink with different concentrations.

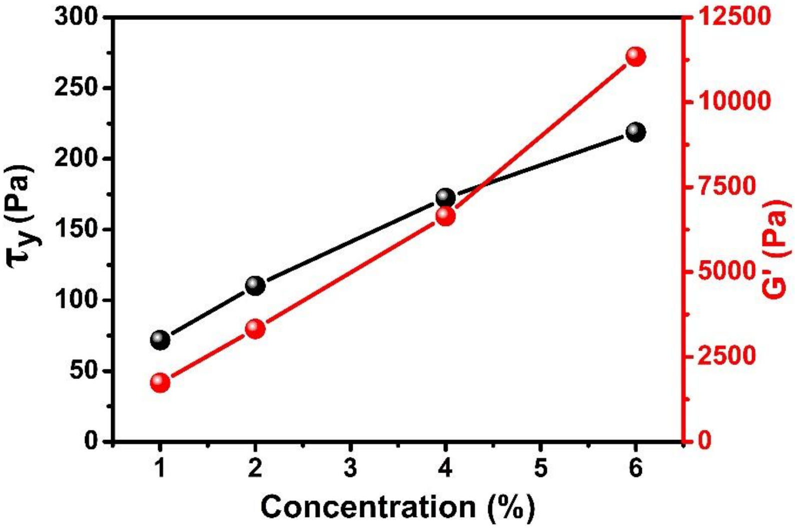

Figure S8. G′ at the plateau region and τ_y of CMU gel inks with different concentrations (Note: The obtained G′ is the average value of G′ within the oscillatory stress range of 0.1-10 Pa in Fig 2d; τ_y is the stress at G′ = G″).

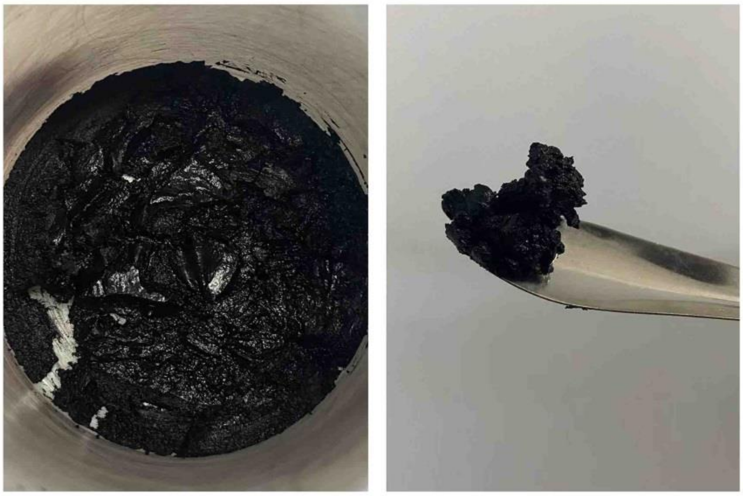

Figure S9. Digital image of the 7 wt% CMU gel ink, exhibiting poor uniformity.

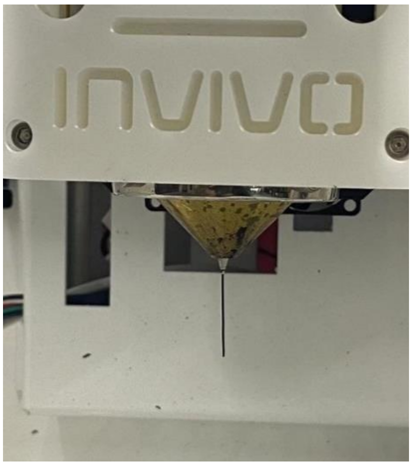

Figure S10. The appearance of extruded highly viscoelastic filament.

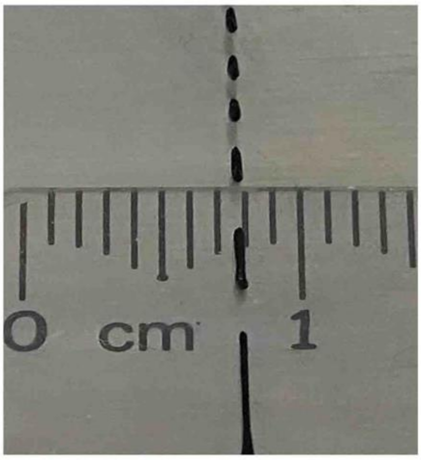

Figure S11. The appearance of extruded discontinuous filament at a high printing speed of 6 $mm{\cdot}s^{-1}$.

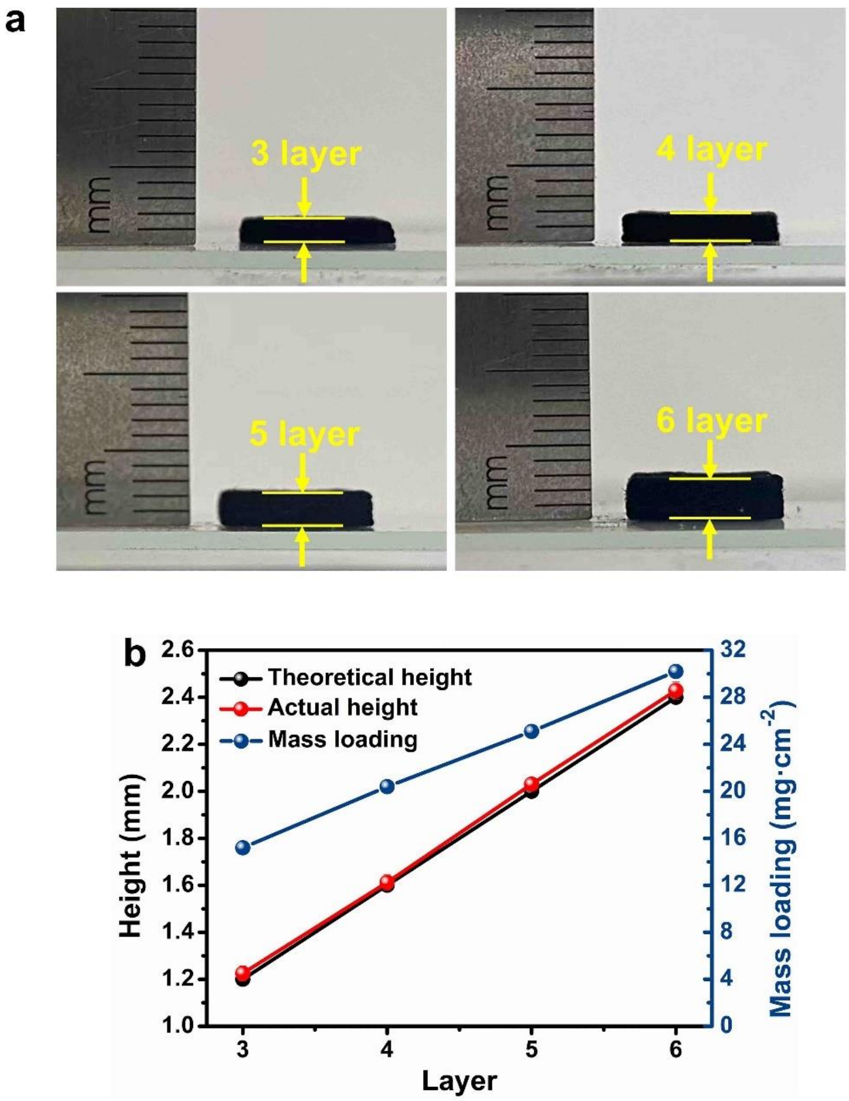

Figure S12. The theoretical height, actual height, and mass loading of the 3D printed, freeze-dried, and annealed block with different layers.

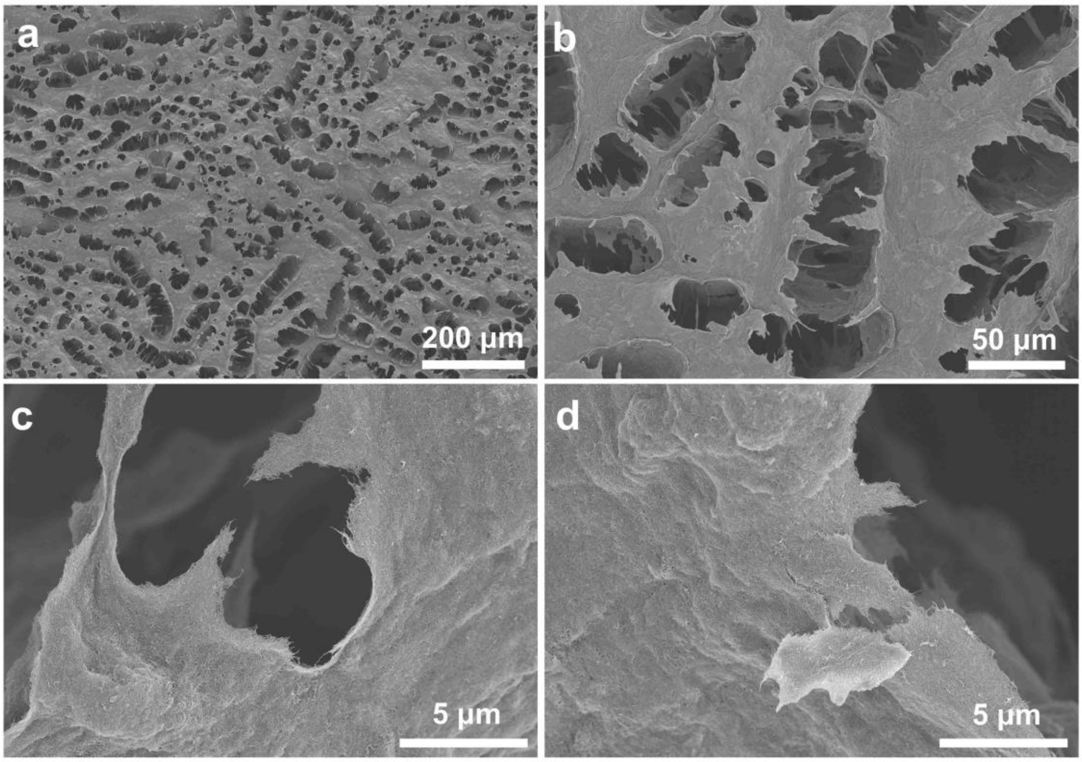

Figure S13. Surface view SEM images of the 6-layer block at different magnifications.

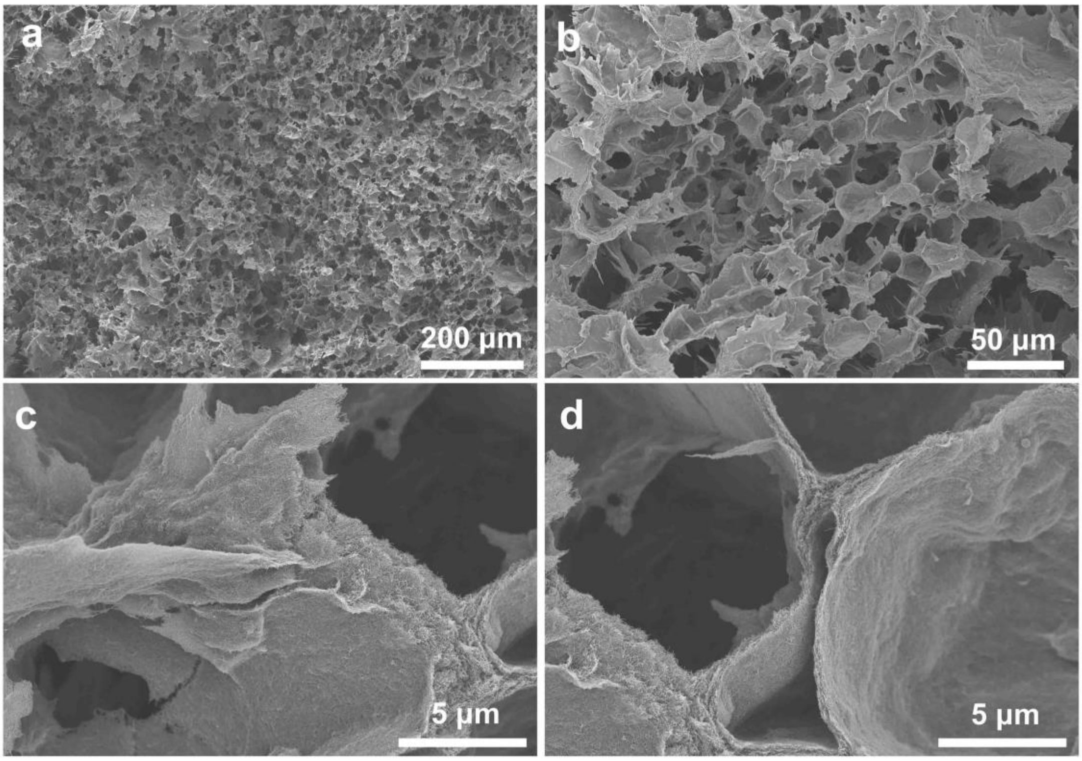

Figure S14. Cross-sectional view SEM images of the 6-layer block at different magnifications.

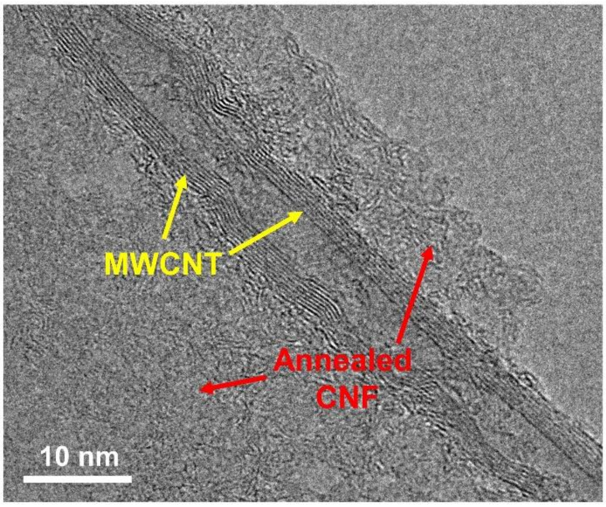

Figure S15. High-resolution TEM image of the 6-layer block.

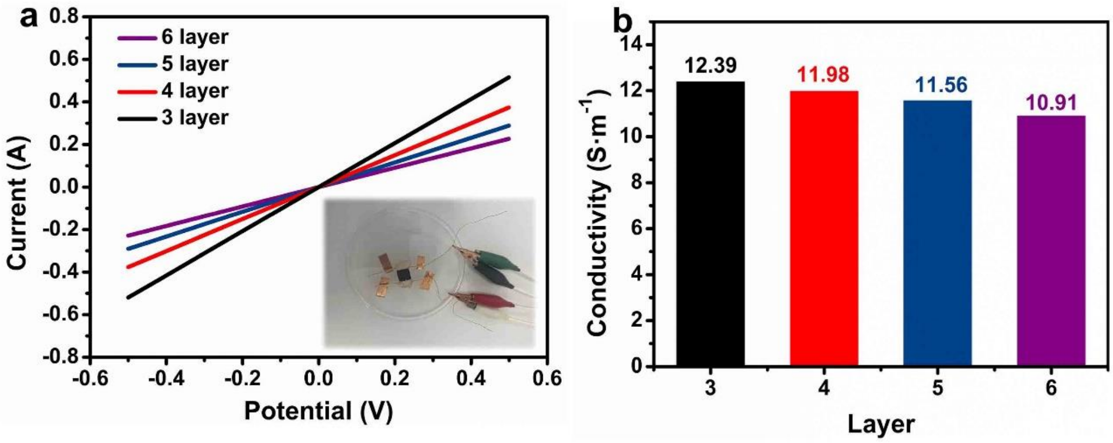

Figure S16. a) Linear voltammetry sweep line of the block with different layers, inset is the experimental setup. b) Conductivity calculated according to equation S2.

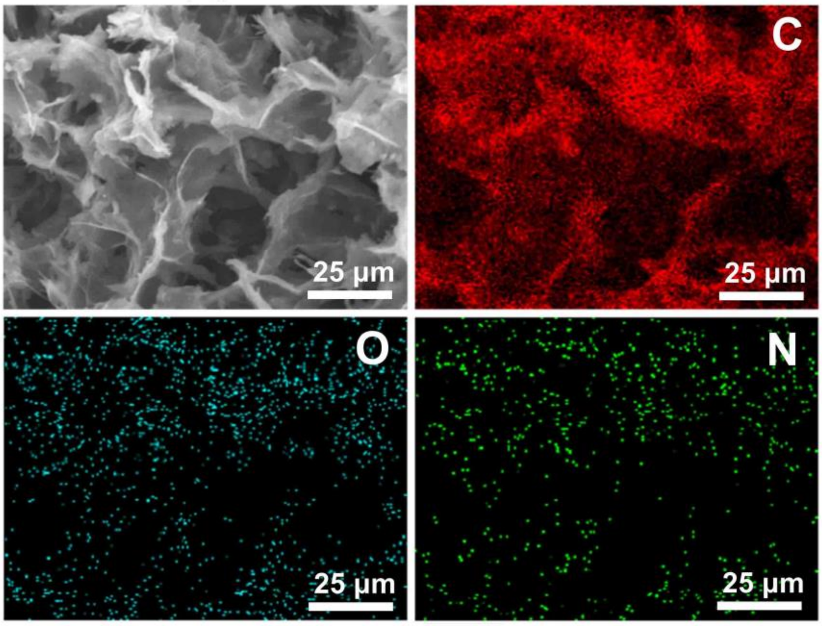

Figure S17. EDS mapping images of the inside morphology of the 6-layer block.

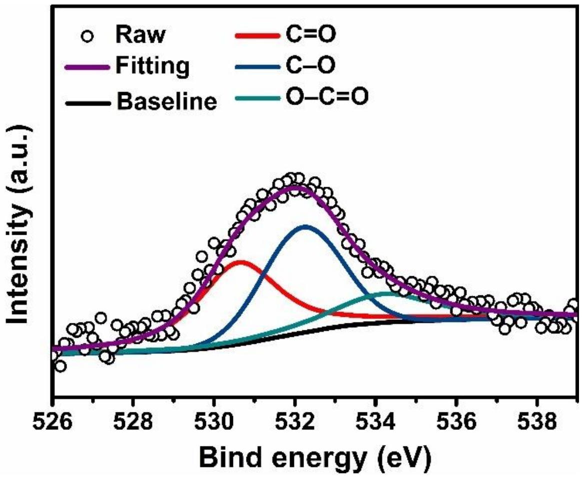

Figure S18. High-resolution O 1s XPS spectra of the 6-layer block.

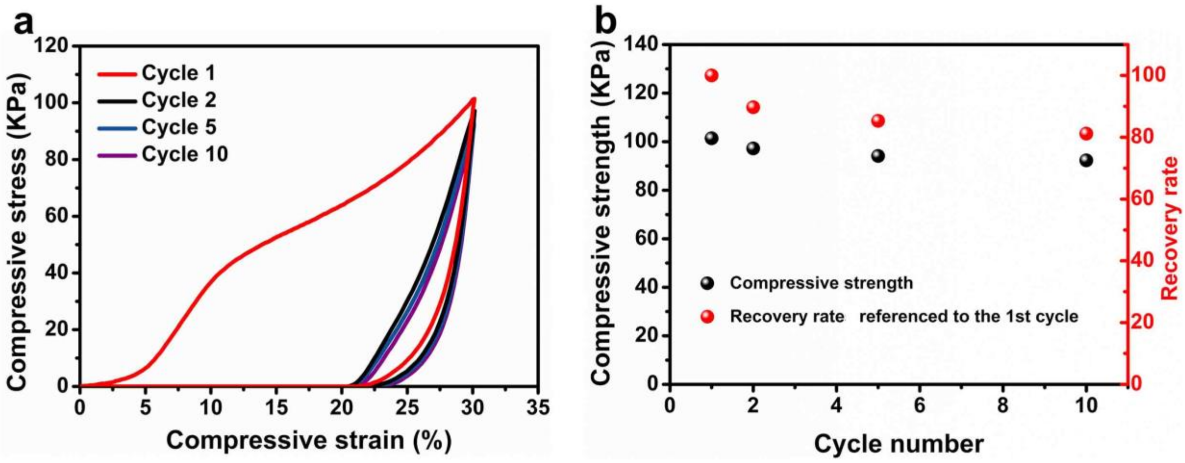

Figure S19. a) Cyclic compressive stress-strain curves of the 3D printed carbon block (6-layer). b) Compressive strength in different cycles and recovery rate referenced to the first cycle after different cycles.

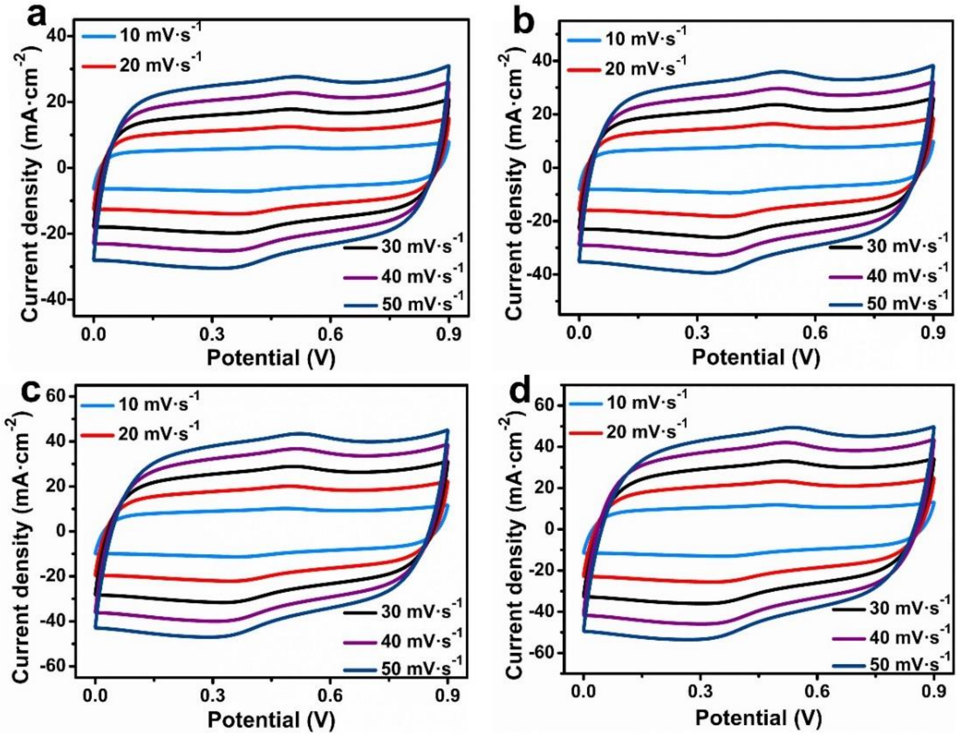

Figure S20. CV profiles of blocks with a) 3, b) 4, c) 5, and d) 6 layers in a three-electrode configuration.

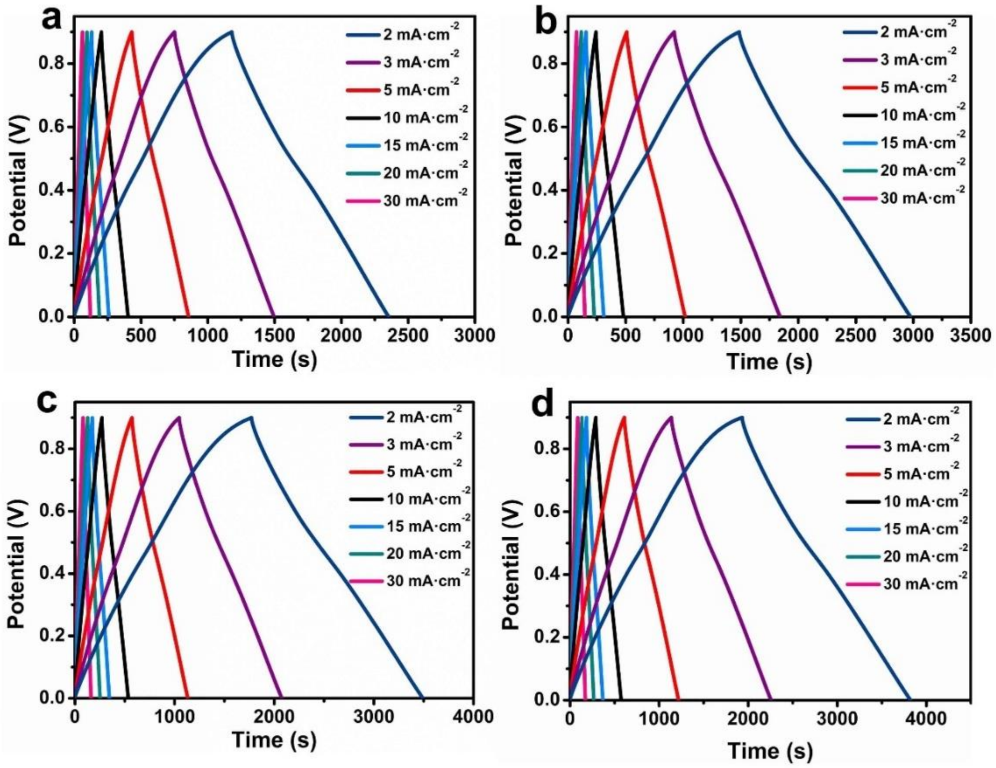

Figure S21. GCD curves of blocks with a) 3, b) 4, c) 5, and d) 6 layers in a three-electrode configuration.

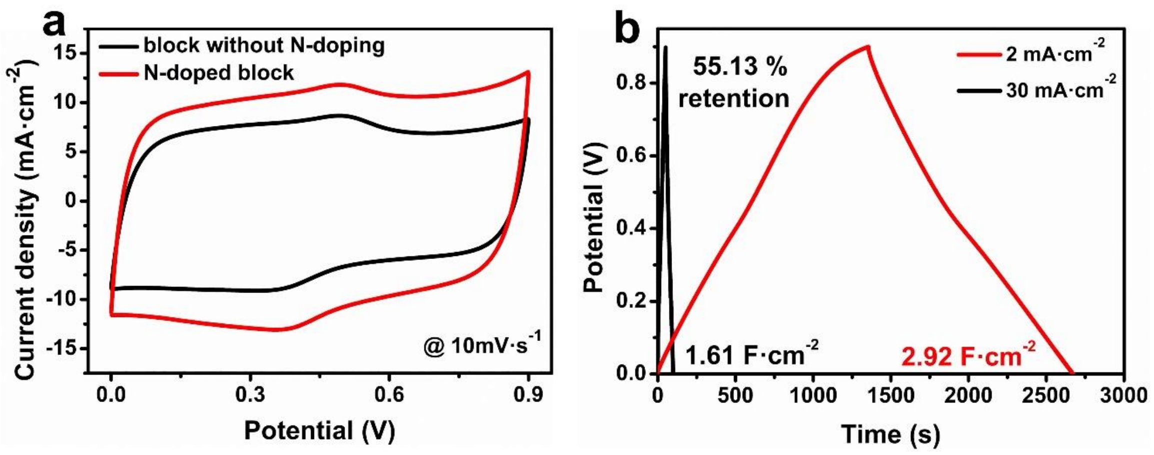

Figure S22. a) CV profiles of the 6-layer blocks with/without N-doping. b) GCD curves of the 6-layer block without N-doping.

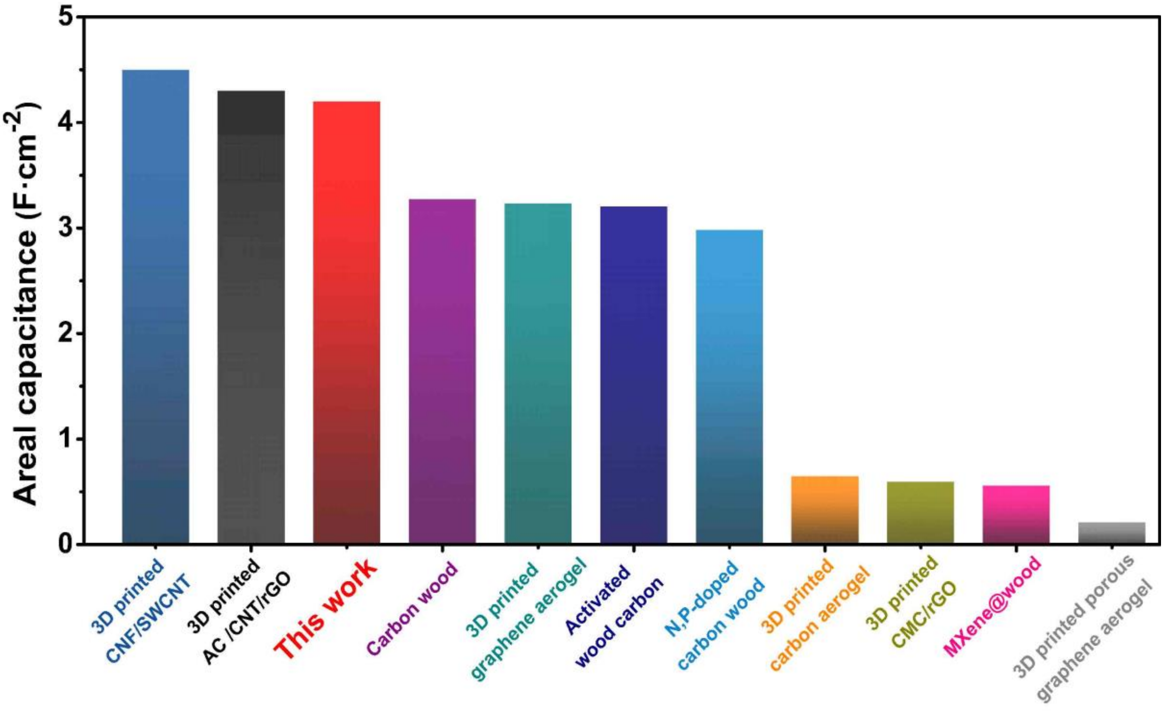

Figure S23. Comparison of the areal capacitance of our 3D printed carbon block (6 layer, 2.43 mm in thickness) with the other thick electrodes, including 3D printed CNF/SWCNT (~4.5 mm in thickness),[3] 3D printed AC/CNT/rRO (~3 mm in thickness),[4] carbon wood (0.68 mm in thickness),[5] 3D printed graphene aerogel (4 mm in thickness),[6] activated wood carbon (0.75 mm in thickness),[7] N,P-doped carbon wood (2.4 mm in thickness),[8] 3D printed carbon aerogel (1-3 mm in thickness),[9] 3D printed CMC/RGO (2 layer, unclear thick),[10] MXene@wood (0.5 mm in thickness),[11] 3D printed porous graphene aerogel (1 mm in thickness).[12]

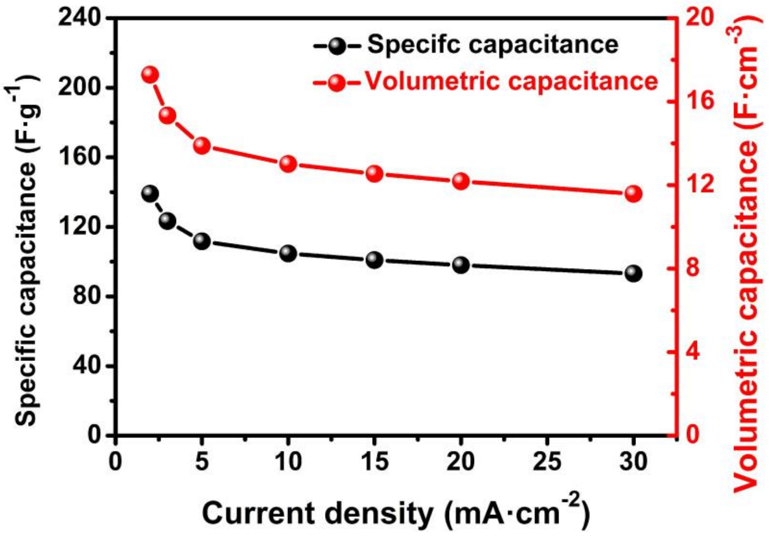

Figure S24. The specific and volumetric capacitance of the 6-layer block at various current densities.

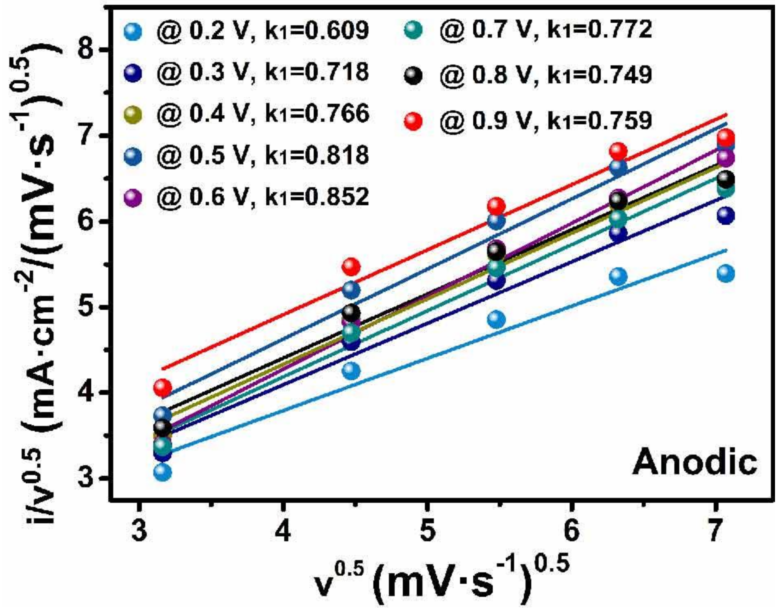

Figure S25. Linear relationship between the anodic current response to the square root of the scan rate and square root of scan rate at a given potential.

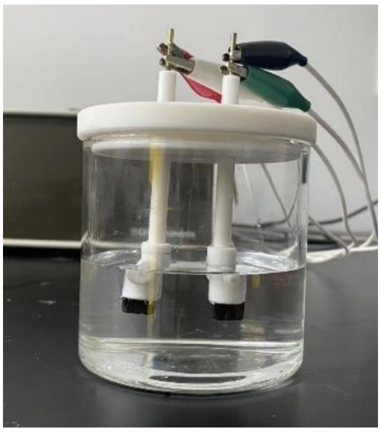

Figure S26. The digital image of the two-electrode system.

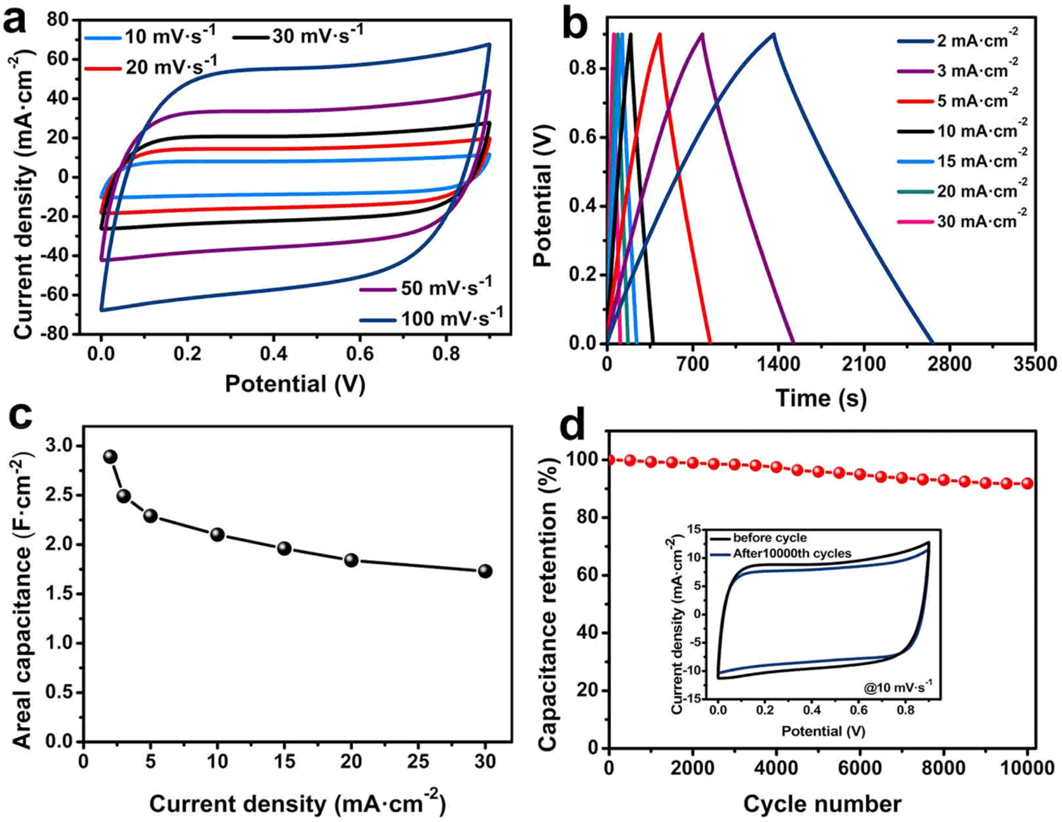

Figure S27. a) CV profiles, and b) GCD curves of the SC. c) The area capacitance, and d) capacitance retention measured for 10000 cycles at a current density of 30 mA·cm^{-2}, inset is the CV profiles at 10 mV·s^{-1} before and after cycling.

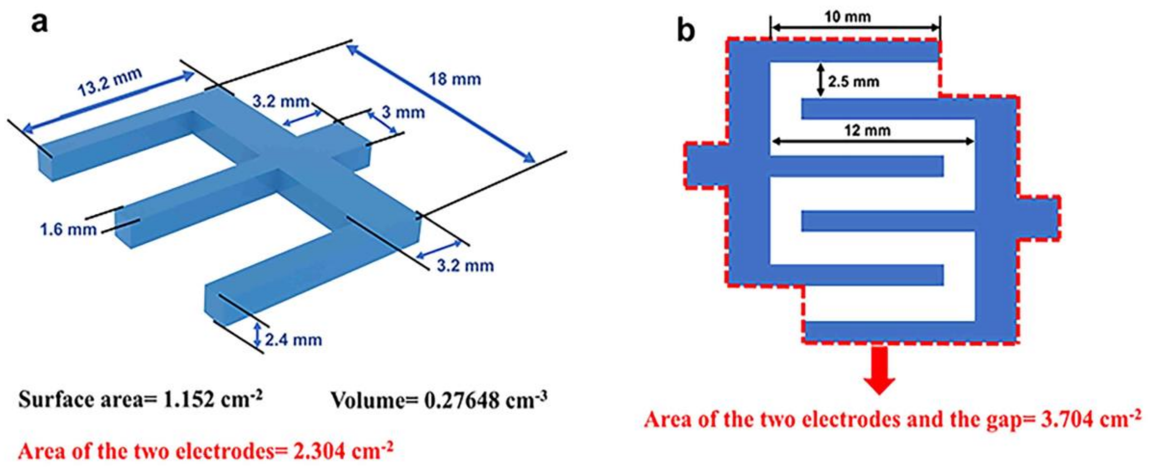

Figure S28. a) The dimension parameters of the interdigitated model, and the area of the two electrodes is 2.304 cm^{-2}. b) The dimension parameters of the two electrodes and their gap, and the area of the two electrodes and the gap is 3.704 cm^{-2}.

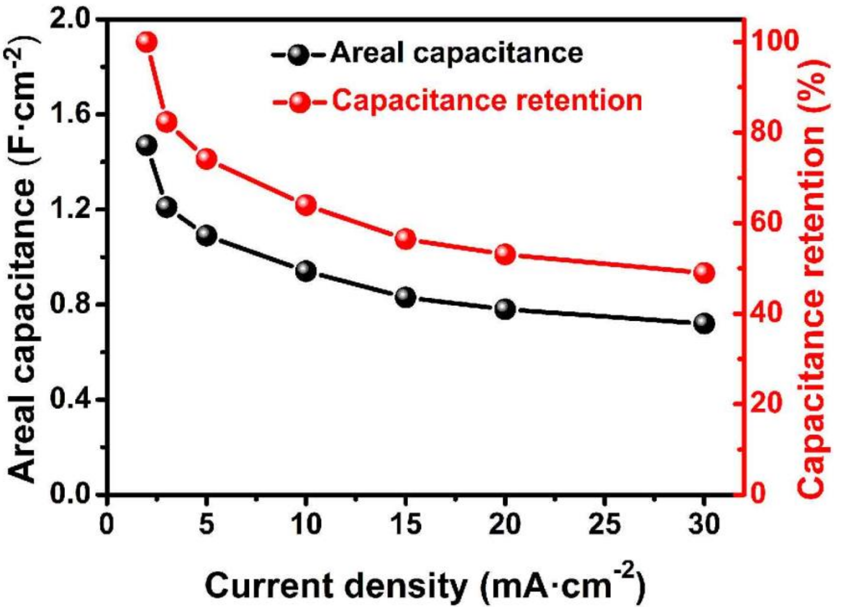

Figure S29. The areal capacitance and capacitance retention of 3D printed QSSC device at different current densities based on the area of two electrodes (2.304 cm^{-2}).

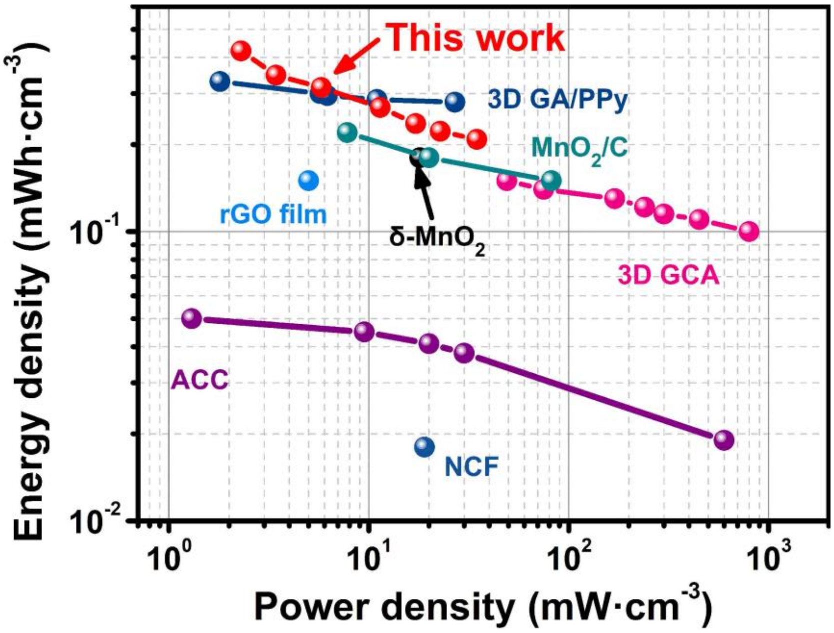

Figure S30. Volumetric Ragone plot of 3D printed QSSC device with the previously reported devices, including 3D GA/PPy,[13] 3D GCA,[14] and ACC,[15] rGO film,[16] δ-MnO_2,[17] MnO_2/C,[18] and NCF.[19]

Tables

Table S1. The calculated consistency coefficient (*K*), flow behavior index (*n*), and R^2 for the CMU gel inks with different concentrations using the Power law.

Ink concentration	Consistency coefficient (*K*)	Flow behavior index (*n*)	R^2
1 wt%	99.85	0.179	0.95
2 wt%	182.74	0.115	0.96
4 wt%	285.89	0.087	0.97
6 wt%	387.09	0.084	0.93

Table S2. The G′ at the plateau region and yield stress of the CMU gel inks with different concentrations.

Ink concentrations	Yield stress/Pa	G′ at the plateau region/Pa
1 wt%	71.8	1733.53
2 wt%	110.2	3320.68
4 wt%	172.34	6641.84
6 wt%	218.89	11345.14

Table S3. The critical radius (r_c), fluid range (i.e., fluid range), and gel range obtained through theoretical analysis. Note: d, r, and L are the diameter, radius, and length of the printed nozzle, respectively; ΔP is the applied air pressure.

d (μm)	r=d/2 (μm)	L (mm)	ΔP (KPa)	Formula	r_c (mm)	Fluid range (vol%)	Gel range (vol%)
400	200	2	45	$\tau(r) = \Delta P \cdot r \cdot (2L)^{-1}$	0.0195	90.25	9.75

Table S4. The height, area, and mass loading of the 3d printed, freeze-dried, and annealed block with different layers.

Layer number	Height/mm	area/cm^2	mass loading/$mg \cdot cm^{-2}$
3	1.22	1	15.2
4	1.61	1	20.4
5	2.03	1	25.1
6	2.43	1	30.2

Table S5. Comparison of the areal capacitance of our developed QSSC device with those of the previously reported devices.

SC type	Electrolyte	Areal capacitance	Reference
**3D printed carbon block**	**PVA/H_2SO_4**	**0.91** F·cm^{-2} @ 2 mA·cm^{-2}	**This work**
**3D printed rGO**	H_2SO_4	0.639 F·cm^{-2} @ 4 mA·cm^{-2}	[20]
**3D printed rGO/CMC**	PVA/H_2SO_4	0.184 F·cm^{-2} @ 3 mA·cm^{-2}	[10]
**CNC/MWCNT/PPy**	PVA/Na_2SO_4	0.54 F·cm^{-2} @ 2 mV·s^{-1}	[21]
**N-doped carbon nanofiber aerogel**	PVA/KOH	0.225 F·cm^{-2} @ 0.25 A·g^{-1}	[22]
**rGO-MWNTMS**	PVA/H_3PO_4	0.118 F·cm^{-2} @ 0.2 A·g^{-1}	[23]
**MWCNTS @ Ni-coated cotton fabric**	PVA/LiCl	0.275 F·cm^{-2} @ 1 mA·cm^{-2}	[24]
**PPy/BPO-CNT**	PVA/H_3PO_4	0.165 F·cm^{-2} @ 0.2 mA·cm^{-2}	[25]
**3D printed carbon aerogel**	KOH	0.231 F·cm^{-2} @ 1.2 mA·cm^{-2}	[26]
**Carbon wood**	KOH	0.846 F·cm^{-2} @ 1 mA·cm^{-2}	[27]
**3D printed rGO aerogel**	PVA/LiOH	-	[28]
**$Ti_3C_2T_x$ /graphene**	PVA/H_3PO_4	0.372 F·cm^{-2} @ 20 mV·s^{-1}	[29]
**$Ti_3C_2T_x$**	PVA/KOH	-	[30]
**PANi/Rgo/Fabric**	PAM/BC/ H_2SO_4	0.564 F·cm^{-2} @ 1 mA·cm^{-2}	[31]
**N, P doped carbon wood**	PVA/H_2SO_4	1.363 F·cm^{-2} @ 1 mA·cm^{-2}	[32]

Table S6. Comparison of the energy density and power density of our developed QSSC device with those of the previously reported devices.

SC type	Electrolyte	Voltage (V)	Energy density ($mWh\cdot cm^{-2}$)	Power density ($mW\cdot cm^{-2}$)	Reference
**3D printed carbon block**	**PVA/H_2SO_4**	**0-0.9**	**0.10**	**0.56**	**This work**
**3D printed MXene/CNF**	PVA/H_2SO_4	0-0.6	0.101	0.299	[2b]
**3D printed rGO**	KOH	0-0.8	0.053	1.54	[20]
**3D printed rGO/CMC**	LiOH	0-1	0.0512	0.968	[10]
**VN/CNT fiber**	PVA/Na_2SO_4	0-1.6	0.096	0.27	[33]
**rGO/PEDOT:PSS**	Na_2SO_4	0-0.8	0.0271	0.0665	[34]
**Coaxial all-carbon fiber**	PVA/H_3PO_4	0-1	0.0098	0.1894	[35]
**3D printed MXene**	PVA/H_2SO_4	0-0.6	0.0244	0.64	[36]
**3D printed MXene**	PVA/H_2SO_4	0-0.6	0.0517	5.7	[2a]
**MXene fiber**	PVA/H_2SO_4	0-0.4	0.0073	0.132	[37]
**Stamped MXene**	PVA/H_2SO_4	0-0.6	0.00072	0.33	[38]
**N-CNFs/rGO/BC**	KOH	0-1	0.11	1	[39]
**rGO/PANi**	PVA/H_2SO_4	0-0.8	0.14	0.27	[40]
**PANi/PCH**	PVA/H_2SO_4	0-0.8	0.042	0.16	[41]
**PVA/PEDOT:PSS**	PVA/H_2SO_4	0-0.8	0.01146	0.2005	[42]
**PVA/PANi**	PVA/Glycerol/H_2SO_4	0-0.8	0.0143	0.0975	[43]
**PEDOT:PSS @ cellulose fabric**	sweat solution	0-0.8	0.00136	0.4	[44]

Reference

[1] G. Zhou, X. Wang, T. Wan, C. Liu, W. Chen, S. Jiang, J. Han, Y. Yan, M. C. Li, C. Mei, *Energy. Environ. Mater.* e12454.

[2] a) J. Orangi, F. Hamade, V. A. Davis, M. Beidaghi, *Acs. Nano.* **2020**, 14, 640; b) G. Zhou, M. C. Li, C. Liu, Q. Wu, C. Mei, *Adv. Funct. Mater.* **2022**, 32, 2109593.

[3] W. Kang, L. Zeng, S. Ling, C. Zhang, *Adv. Energy. Mater.* **2021**, 11, 2100020.

[4] T. Gao, Z. Zhou, J. Yu, J. Zhao, G. Wang, D. Cao, B. Ding, Y. Li, *Adv. Energy. Mater.* **2019**, 9, 1802578.

[5] Y. M. Wang, X. J. Lin, T. Liu, H. Chen, S. Chen, Z. J. Jiang, J. Liu, J. L. Huang, M. L. Liu, *Adv. Funct. Mater.* **2018**, 28.

[6] B. Yao, S. Chandrasekaran, H. Z. Zhang, A. Ma, J. Z. Kang, L. Zhang, X. H. Lu, F. Qian, C. Zhu, E. B. Duoss, C. M. Spadaccini, M. A. Worsley, Y. Li, *Adv. Mater.* **2020**, 32.

[7] C. J. Chen, Y. Zhang, Y. J. Li, J. Q. Dai, J. W. Song, Y. G. Yao, Y. H. Gong, I. Kierzewski, J. Xie, L. B. Hu, *Energ. Environ. Sci.* **2017**, 10, 538.

[8] K. Liu, R. W. Mo, W. J. Dong, W. Zhao, F. Q. Huang, *J. Mater. Chem. A.* **2020**, 8, 20072.

[9] S. Chandrasekaran, B. Yao, T. Y. Liu, W. Xiao, Y. Song, F. Qian, C. Zhu, E. B. Duoss, C. M. Spadaccini, Y. Li, M. A. Worsley, *Mater. Horiz.* **2018**, 5, 1166.

[10] S. Tagliaferri, G. Nagaraju, A. Panagiotopoulos, M. Och, G. Cheng, F. Iacoviello, C. Mattevi, *Acs. Nano.* **2021**, 15, 15342.

[11] M. Luo, D. Zhang, K. Yang, Z. Li, Y. Hu, S. Xia, W. Chen, X. Zhou, *Fuel. Process. Technol.* **2022**, 238, 107496.

[12] C. Zhu, T. Y. Liu, F. Qian, T. Y. J. Han, E. B. Duoss, J. D. Kuntz, C. M. Spadaccini, M. A. Worsley, Y. Li, *Nano. Lett.* **2016**, 16, 3448.

[13] Z. Qi, J. Ye, W. Chen, J. Biener, E. B. Duoss, C. M. Spadaccini, M. A. Worsley, C. Zhu, *Adv. Mater. Technol*-us **2018**, 3, 1800053.

[14] Y. Song, T. Y. Liu, B. Yao, T. Y. Kou, D. Y. Feng, X. X. Liu, Y. Li, *Small.* **2017**, 13, 1700067.

[15] J. Wang, J. Polleux, J. Lim, B. Dunn, *The Journal of Physical Chemistry C* **2007**, 111, 14925.

[16] Z. S. Wu, K. Parvez, X. Feng, K. Müllen, *Nat. Commun.* **2013**, 4, 1.

[17] Y. Wang, Y.-Z. Zhang, D. Dubbink, E. Johan, *Nano. Energy.* **2018**, 49, 481.

[18] X. Xiao, T. Li, P. Yang, Y. Gao, H. Jin, W. Ni, W. Zhan, X. Zhang, Y. Cao, J. Zhong, *Acs. Nano.* **2012**, 6, 9200.

[19] K. Xiao, L. X. Ding, G. Liu, H. Chen, S. Wang, H. Wang, *Adv. Mater.* **2016**, 28, 5997.

[20] X. Tang, H. Zhou, Z. Cai, D. Cheng, P. He, P. Xie, D. Zhang, T. Fan, *Acs. Nano.* **2018**, 12, 3502.

[21] K. Shi, X. Yang, E. D. Cranston, I. Zhitomirsky, *Adv. Funct. Mater.* **2016**, 26, 6437.

[22] H. Chen, T. Liu, J. Mou, W. Zhang, Z. Jiang, J. Liu, J. Huang, M. Liu, *Nano. Energy.* **2019**, 63, 103836.

[23] S. Gao, K. Wang, Z. Du, Y. Wang, A. Yuan, W. Lu, L. Chen, *Carbon.* **2015**, 92, 254.

[24] Q. Huang, L. Liu, D. Wang, J. Liu, Z. Huang, Z. Zheng, *J. Mater. Chem. A.* **2016**, 4, 6802.

[25] Z. Lv, Y. Tang, Z. Zhu, J. Wei, W. Li, H. Xia, Y. Jiang, Z. Liu, Y. Luo, X. Ge, *Adv. Mater.* **2018**, 30, 1805468.

[26] S. Yuan, W. Fan, D. Wang, L. Zhang, Y.-E. Miao, F. Lai, T. Liu, *J. Mater. Chem. A.* **2021**, 9, 423.

[27] F. Wang, L. Zhang, Q. Zhang, J. Yang, G. Duan, W. Xu, F. Yang, S. Jiang, *Applied. Energy.* **2021**, 289, 116734.

[28] C. Zhu, T. Liu, F. Qian, T. Y.-J. Han, E. B. Duoss, J. D. Kuntz, C. M. Spadaccini, M. A. Worsley, Y. Li, *Nano. Lett.* **2016**, 16, 3448.
[29] Q. Yang, Z. Xu, B. Fang, T. Huang, S. Cai, H. Chen, Y. Liu, K. Gopalsamy, W. Gao, C. Gao, *J. Mater. Chem. A.* **2017**, 5, 22113.
[30] S. Xu, G. Wei, J. Li, Y. Ji, N. Klyui, V. Izotov, W. Han, *Chem Eng J* **2017**, 317, 1026.
[31] X. Li, L. Yuan, R. Liu, H. He, J. Hao, Y. Lu, Y. Wang, G. Liang, G. Yuan, Z. Guo, *Adv. Energy. Mater.* **2021**, 11, 2003010.
[32] K. Liu, R. Mo, W. Dong, W. Zhao, F. Huang, *J. Mater. Chem. A.* **2020**, 8, 20072.
[33] Q. Zhang, X. Wang, Z. Pan, J. Sun, J. Zhao, J. Zhang, C. Zhang, L. Tang, J. Luo, B. Song, *Nano. Lett.* **2017**, 17, 2719.
[34] G. Qu, J. Cheng, X. Li, D. Yuan, P. Chen, X. Chen, B. Wang, H. Peng, *Adv. Mater.* **2016**, 28, 3646.
[35] V. T. Le, H. Kim, A. Ghosh, J. Kim, J. Chang, Q. A. Vu, D. T. Pham, J.-H. Lee, S.-W. Kim, Y. H. Lee, *Acs. Nano.* **2013**, 7, 5940.
[36] W. Yang, J. Yang, J. J. Byun, F. P. Moissinac, J. Xu, S. J. Haigh, M. Domingos, M. A. Bissett, R. A. Dryfe, S. Barg, *Adv. Mater.* **2019**, 31, 1902725.
[37] M. Hu, Z. Li, G. Li, T. Hu, C. Zhang, X. Wang, *Adv. Mater. Technol*-us. **2017**, 2, 1700143.
[38] C. Zhang, M. P. Kremer, A. Seral - Ascaso, S. H. Park, N. McEvoy, B. Anasori, Y. Gogotsi, V. Nicolosi, *Adv. Funct. Mater.* **2018**, 28, 1705506.
[39] L. Ma, R. Liu, H. Niu, L. Xing, L. Liu, Y. Huang, *Acs. Appl. Mater. Inter.* **2016**, 8, 33608.
[40] L. Qin, G. Yang, D. Li, K. Ou, H. Zheng, Q. Fu, Y. Sun, *Chem. Eng. J.* **2022**, 430, 133045.
[41] K. Wang, X. Zhang, C. Li, X. Sun, Q. Meng, Y. Ma, Z. Wei, *Adv. Mater.* **2015**, 27, 7451.
[42] Q. Liu, J. Qiu, C. Yang, L. Zang, G. Zhang, E. Sakai, *Adv. Mater. Technol*-us. **2021**, 6, 2000919.
[43] F. Lai, Z. Fang, L. Cao, W. Li, Z. Lin, P. Zhang, *Ionics.* **2020**, 26, 3015.
[44] L. Manjakkal, A. Pullanchiyodan, N. Yogeswaran, E. S. Hosseini, R. Dahiya, *Adv. Mater.* **2020**, 32, 1907254.
